# A distinct metabolic response characterizes sensitivity to EZH2 inhibition in multiple myeloma

**DOI:** 10.1038/s41419-021-03447-8

**Published:** 2021-02-12

**Authors:** Patrick Nylund, Alba Atienza Párraga, Jakob Haglöf, Elke De Bruyne, Eline Menu, Berta Garrido-Zabala, Anqi Ma, Jian Jin, Fredrik Öberg, Karin Vanderkerken, Antonia Kalushkova, Helena Jernberg-Wiklund

**Affiliations:** 1 Science for Life Laboratory, Department of Immunology, Genetics and Pathology, Rudbeck Laboratory, Uppsala University, Uppsala, Sweden; 2 Department of Medicinal Chemistry, Analytical Pharmaceutical Chemistry, Uppsala University, Uppsala, Sweden; 3 Department of Haematology and Immunology-Myeloma Center Brussels, Vrije Universiteit Brussel (VUB), Brussels, Belgium; 4 Mount Sinai Center for Therapeutics Discovery, Departments of Pharmacological Sciences and Oncological Sciences, Tisch Cancer Institute, Icahn School of Medicine at Mount Sinai, New York, NY 10029 USA

**Keywords:** Cancer epigenetics, Cancer metabolism

## Abstract

Multiple myeloma (MM) is a heterogeneous haematological disease that remains clinically challenging. Increased activity of the epigenetic silencer EZH2 is a common feature in patients with poor prognosis. Previous findings have demonstrated that metabolic profiles can be sensitive markers for response to treatment in cancer. While EZH2 inhibition (EZH2i) has proven efficient in inducing cell death in a number of human MM cell lines, we hereby identified a subset of cell lines that despite a global loss of H3K27me3, remains viable after EZH2i. By coupling liquid chromatography-mass spectrometry with gene and miRNA expression profiling, we found that sensitivity to EZH2i correlated with distinct metabolic signatures resulting from a dysregulation of genes involved in methionine cycling. Specifically, EZH2i resulted in a miRNA-mediated downregulation of methionine cycling-associated genes in responsive cells. This induced metabolite accumulation and DNA damage, leading to G2 arrest and apoptosis. Altogether, we unveiled that sensitivity to EZH2i in human MM cell lines is associated with a specific metabolic and gene expression profile post-treatment.

## Introduction

Multiple myeloma (MM) is a genetically and clinically heterogeneous haematological disease, characterized by a monoclonal expansion of malignant plasmablasts/plasma cells in the bone marrow^1–3^. A complex mutational landscape, high heterogeneity and a genome-wide epigenetic reconfiguration may contribute to the underlying causes of drug resistance and relapse^4–6^.

We have previously demonstrated that the gene silencing profile of MM cells is reminiscent of the one of embryonic fibroblasts, where the Polycomb repressive complex 2 (PRC2) is highly active^7^. Moreover, an increased deposition of histone H3 lysine 27 trimethylation (H3K27me3) by PRC2 correlates with advanced stages of the disease^4^ and overexpression of the catalytic subunit of PRC2, enhancer of zeste homologue 2 (EZH2), is a common feature of MM^8,9^. These data support the notion that PRC2-mediated silencing is a key mechanism in MM and may represent a suitable drug target^4,10^. In line with this, selective inhibition of EZH2 (EZH2i) with UNC1999 impaired cell viability and induced apoptosis in MM cell lines and primary patient samples^11^, and upregulated tumour suppressor miRNAs that repress oncogenes with a vital function for MM cell growth and survival^11–13^.

Metabolic profiles are altered upon drug treatment, thus panels of metabolites have been used as biomarkers for drug response^14,15^. The association between the metabolome and the epigenome is, however, complex. It is well known that chromatin regulators require metabolite intermediates as cofactors and that different metabolic pathways associated with tumorigenesis are epigenetically regulated^16^. However, the impact of epigenetic targeting on the cellular metabolome is largely unexplored.

In this study, we found that in vivo EZH2i with UNC1999 effectively reduced tumour load in the 5T33MM syngeneic murine model, further emphasising the potential of EZH2 as a target for clinical intervention. However, when exploring the effects of EZH2i in an extended panel of human MM cell lines, we observed that a subset of cell lines remained viable after the global loss of H3K27me3. Combining liquid chromatography-mass spectrometry (LC-MS), chromatin immunoprecipitation (ChIP), gene and miRNA expression profiling, we identified distinct alterations in the methionine cycling pathways in cell lines that responded to EZH2i, which were absent in non-responsive cell lines. These metabolic alterations e.g., homocysteine and 5-methyltetrahydrofolic acid accumulation, correlated with the upregulation of EZH2-targeted miRNAs (e.g., miR-494-3p, miR-130a-3p, miR-134-5p and miR-192-5p), which in turn downregulated genes involved in the methionine and homocysteine degradation pathways (e.g., *MAT2A/2B*, *CBS* and *CTH*). This was associated with DNA double-strand breaks, G2 arrest and apoptosis. Altogether, our study unveils a mechanism of clinical relevance that underlies responsiveness to EZH2i in human MM cell lines.

## Results

### UNC1999 reduced tumour burden in the 5T33MM murine model

**Fig. 1:**
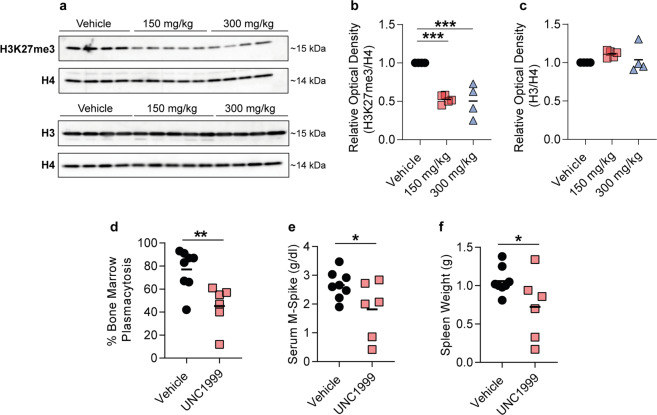
EZH2 inhibition significantly reduced tumour burden in mice bearing 5T33MM multiple myeloma. **a** Western blot against H3K27me3 and total histone H3 (H3) of bone marrow plasma cells derived from 5T33MM mice treated with 150 or 300 mg/kg of UNC1999, or with vehicle. Total histone H4 (H4) was used as a loading control. Corresponding uncropped western blots can be found in Supplementary Fig. 9a–b. **b** Signal quantification of the western blot against H3K27me3 shown in (**a**). **c** Signal quantification of the western blot against H3 shown in (**a**). The optical density in (**b**–**c**) was normalized against H4. *n*_(Vehicle)_ = 4, *n*_(150 mg/kg)_ = 5 and *n*_(300 mg/kg)_ = 4. Statistical analysis in (**b**–**c**) was performed with one-way ANOVA. Values: mean with SEM. **d**–**f** 5T33MM mice were treated with 200 mg/kg of UNC1999 or with vehicle. Tumour load was determined by **d** bone marrow plasmacytosis, **e** M-spike levels and **f** spleen weight. *n*_(Vehicle)_ = 8, *n*_(UNC1999)_ = 6. Statistical analysis in (**d**–**f**) was performed with one-sided *t*-test. Values: mean with SEM. *p < 0.05, **p < 0.01, ***p < 0.001, ****p < 0.0001.

To test the effects of EZH2i in vivo, we administered the EZH2 inhibitor UNC1999 to 5T33MM mice, which share characteristics with human MM^17,18^. C57BL/KaLwRij^19^ mice were injected with 5T33MM cells and treated with UNC1999. To ensure on-target effects of UNC1999 in vivo, we analysed H3K27me3 levels in bone marrow plasma cells from 5T33MM mice treated with 150 mg/kg and 300 mg/kg UNC1999 for seven days. We found a global reduction of H3K27me3 in UNC1999-treated mice, which was not due to a loss of total histone H3 (Fig. 1a–c), suggesting that UNC1999 effectively inhibited EZH2 in the bone marrow microenvironment. To evaluate its effects on the tumour burden in vivo, mice were treated with 200 mg/kg of UNC1999 or vehicle until the control group indicated signs of morbidity. We observed a decrease in bone marrow plasmacytosis, spleen weight^20^ and M-spike levels in the serum of treated mice (Fig. 1d–f). Taken together, we observed a positive treatment response to UNC1999 in the 5T33MM preclinical murine model, which encouraged further investigation into an extended panel of human MM cell lines.

### EZH2i induced a distinct metabolic signature in responsive MM cell lines

**Fig. 2:**
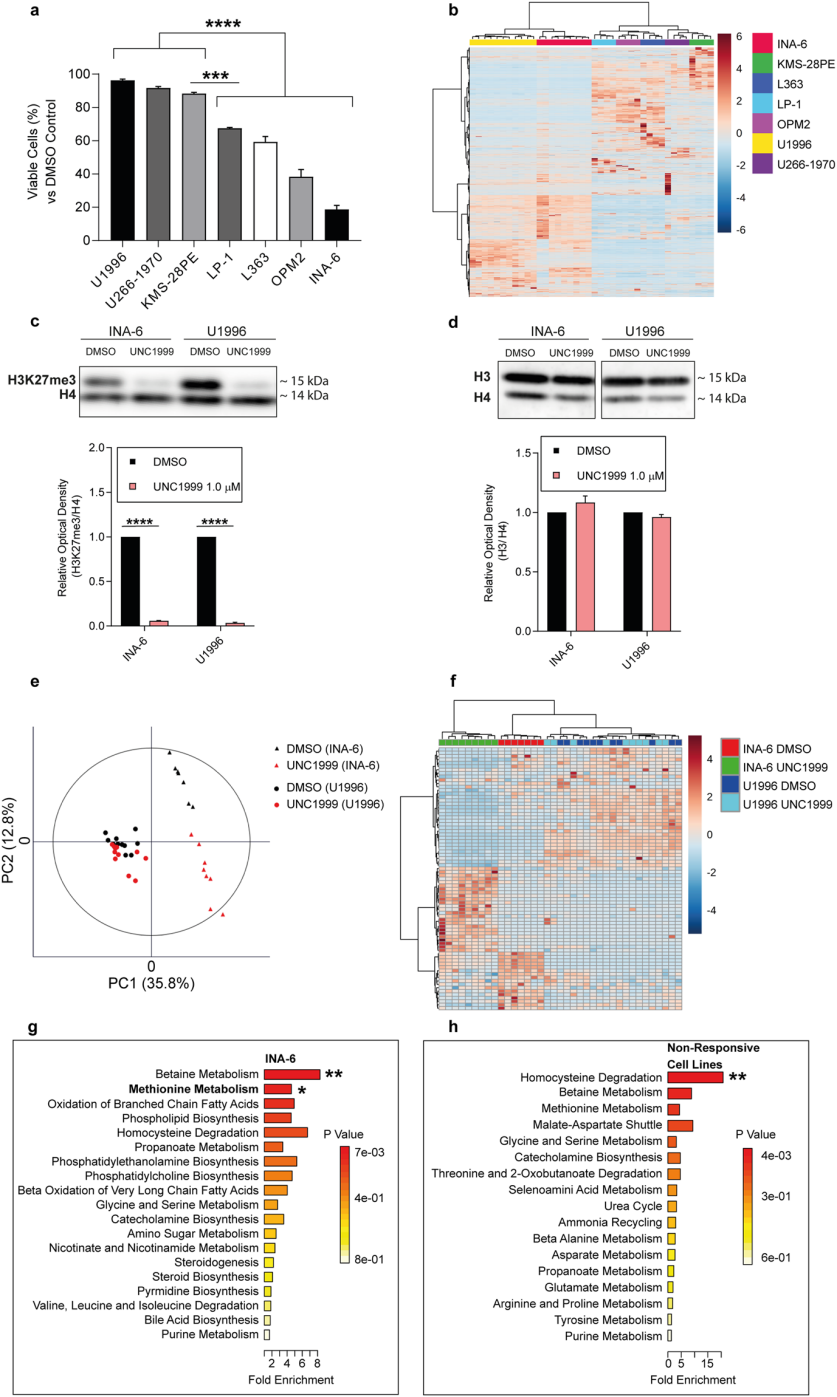
Sensitivity of MM cell lines to UNC1999 treatment was associated with metabolic alterations in methionine metabolism. **a** Effects of UNC1999 treatment on cell viability for the indicated cell lines. Biological replicates = 3. Statistical analysis was performed with one-way ANOVA. Values: mean with SEM. **b** Unsupervised clustering of seven human MM cell lines based on the abundance of all metabolites detected by LC-MS under basal conditions (DMSO treatment only). *n*_(INA-6)_ = 9, *n*_(U1996)_ = 11, *n*_(KMS-28PE, U266-1970, LP-1, L363 and OPM2)_ = 4. **c** Representative western blot against H3K27me3 in the INA-6 (responsive) and U1996 (non-responsive) cells after UNC1999 treatment, and the relative signal quantification. **d** Western blot against H3 in the INA-6 (responsive) and U1996 (non-responsive) cells after UNC1999 treatment, and the relative signal quantification. The optical density in (**d**–**e**) was normalized against H4. Statistical analysis in (**d**–**e**) was performed with a two-tailed *t*-test. Values: mean with SEM. Data were collected from three biological replicates. The corresponding uncropped western blot figures (**d**–**e**) can be found in Supplementary Fig. 9c. **e** A principal component analysis (PCA) demonstrating in two dimensions that non-responding and responding samples cluster based on their independent metabolic profiles. *n*_(DMSO INA-6)_ = 9, *n*_(UNC1999 INA-6)_ = 9, *n*_(DMSO U1996)_ = 11, *n*_(UNC1999 U1996)_ = 10. **f** Heatmap showing the 150 most discriminating metabolites in the dataset, which were significantly different between INA-6 and U1996 after UNC1999 treatment. The colour bar describes the intensity of the change in metabolite levels: deeper colour determines the different magnitude of regulation. Red indicates upregulation and blue indicates downregulation. *n*_(DMSO INA-6)_ = 9, *n*_(UNC1999 INA-6)_ = 9, *n*_(DMSO U1996)_ = 11, *n*_(UNC1999 U1996)_ = 10. **g**–**h** Metabolite set enrichment analysis (MSEA) (**g**) in INA-6 and (**h**) non-responsive cell lines after UNC1999 treatment. *n*_(DMSO INA-6)_ = 9, *n*_(UNC1999 INA-6)_ = 9, *n*_(DMSO KMS-28PE)_ = 4, *n*_(UNC1999 KMS-28PE)_ = 4, *n*_(DMSO U266-1970)_ = 4, *n*_(UNC1999 U266-1970)_ = 4, *n*_(DMSO u1996)_ = 11, *n*_(UNC1999 U1996)_ = 10. *p < 0.05, **p < 0.01, ***p < 0.001, ****p < 0.0001.

We further assessed the response to EZH2i in seven human MM cell lines. Cells were treated with 1 µM of UNC1999 for five days and cell viability was determined at the experimental endpoint. U1996, U266-1970 and KMS-28PE exhibited a non-responsive profile (viability at endpoint > ~80%), whereas OPM2, L363, LP-1, and INA-6 exhibited a responsive profile (<~80% viability) (Fig. 2a and Supplementary Fig. 1a–d).

To investigate the underlying molecular mechanisms in responsive versus non-responsive cells after UNC1999 treatment, all cell lines were subjected to a global metabolic analysis by high-resolution LC-MS after UNC1999 or DMSO treatment. UNC1999 altered the metabolic profile in responsive cell lines to a larger extent than in non-responsive cell lines (Supplementary Fig. 1e–f). Next, we identified differentially abundant metabolites after treatment in responsive (OPM2, L363, LP-1, and INA-6) and non-responsive (U1996, KMS-28PE and U266-1970) cell lines and selected the metabolites with the highest discriminating power for further analyses (Supplementary Fig. 1g). Unsupervised hierarchical clustering based on the identified metabolites revealed that the responsive cell lines OPM2, L363 and LP-1 shared a metabolic signature under basal conditions, which was different from the one of the non-responsive cell lines KMS-28PE and U266-1970 (Fig. 2b). Interestingly, despite having differential viability responses to UNC1999 treatment, U1996 and INA-6 shared a common metabolic profile under basal conditions (Fig. 2b) and EZH2i effectively reduced H3K27me3 levels in both cell lines (Fig. 2c, d). Thus, INA-6 and U1996 were selected for in depth evaluation of changes in metabolite abundance after EZH2i. Principal component analysis (PCA) showed a clear discrimination between INA-6 and U1996 cells upon UNC1999 treatment (Fig. 2e). Based on the top 150 differentially abundant metabolites, a significantly altered metabolic response was observed in INA-6 when treated with UNC1999, while the metabolic profile of U1996 remained unaffected (Fig. 2f). Similar to INA-6, the other responsive cell lines (LP-1, L363 and OPM2) showed a shift in metabolic response as a result of EZH2i (Supplementary Fig. 2a–d). In summary, we identified that sensitivity to EZH2i was associated with altered metabolic response.

### Sensitivity to EZH2i was associated with alterations in the methionine cycling pathways

We next used MetaboAnalyst^21^ to decipher which cellular pathways were involved in the metabolic shift observed in responsive MM cell lines post-UNC1999 treatment. Metabolites highly enriched after UNC1999 treatment were examined by metabolite set enrichment analysis (MSEA). The analysis unveiled a dysregulation of pathways linked to methionine cycling (e.g., betaine metabolism and methionine metabolism) in INA-6 (Fig. 2g), similarly to what was observed for the other responsive cell lines (Supplementary Fig. 2e–g). Due to a lack of sufficient discriminating features in 2/3 resistant cell lines (U266-1970 and U1996), it was not possible to generate individual models, suggesting that metabolic changes induced by EZH2i are associated with sensitivity. Thus, we generated a model that merged the resistant cell lines and found that the only significantly deregulated pathway was homocysteine degradation (Fig. 2h). Importantly, we did not detect major alterations in housekeeping molecules in INA-6 or U1996 cells, suggesting an intact cellular integrity (Supplementary Table I).

**Fig. 3:**
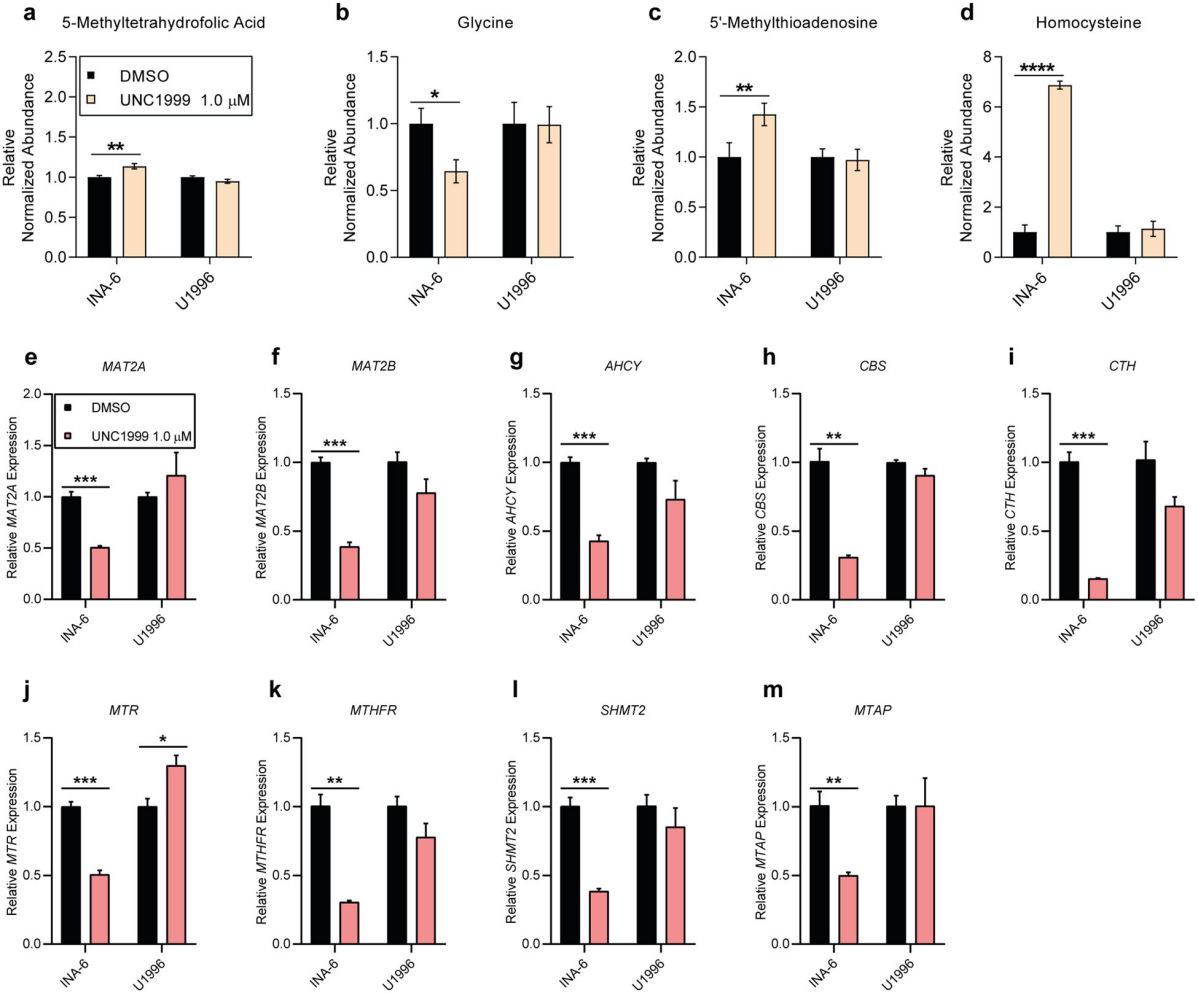
UNC1999 treatment affected metabolites and genes involved in the methionine cycling pathways. **a**–**d** Relative metabolite abundance in INA-6 and U1996 cells after UNC1999 treatment determined by LC-MS for the following metabolites: **a** 5-methyltetrahydrofolic acid, **b** glycine, **c** 5′-methylthioadenosine and **d** homocysteine. *n*_(DMSO INA-6)_ = 9, *n*_(UNC1999 INA-6)_ = 9, *n*_(DMSO U1996)_ = 11, *n*_(UNC1999 U1996)_ = 10. **e**–**m** Gene expression analysis by RT-qPCR of the following methionine cycling-associated genes in INA-6 and U1996 post UNC1999 treatment: **e** *MAT2A*, **f** *MAT2B*, **g** *AHCY*, **h** *CBS*, **i** *CTH*, **j** *MTR*, **k** *MTHFR*, **l** *SHMT2* and **m** *MTAP*. Data were collected from three biological replicates. Statistical analysis was performed with multiple *t*-test. Values: mean with SEM. *p < 0.05, **p < 0.01, ***p < 0.001, ****p < 0.0001.

On a metabolite level, UNC1999-treated INA-6 cells showed an accumulation of 5-methyltetrahydrofolic acid (Fig. 3a), a decrease in glycine abundance (Fig. 3b) and an accumulation of 5-methylthioadenosine (Fig. 3c), while methionine and adenosine remained unaffected (Supplementary Fig. 3j–k). Similar effects were observed in one or more of the other responsive cell lines (Supplementary Fig. 3a–c, e–f). Interestingly, we found a clear accumulation of homocysteine in INA-6 cells after UNC1999 treatment, which was 6.8 times larger than in the DMSO-treated control (Fig. 3d), while the by-products of homocysteine degradation showed no alterations (Supplementary Fig. 3l–n). The other responsive cell lines showed variable responses in terms of accumulation of metabolites involved in homocysteine degradation (Supplementary Fig. 3d, h–i). No significant changes in specific metabolites were observed in the non-responsive cell lines (Fig. 3g–j and Supplementary Fig. 3a–i). Altogether, sensitivity to EZH2i was associated with changes in the abundance of metabolites within the methionine cycling pathways.

### Sensitivity to EZH2i correlated with downregulation of genes involved in methionine cycling

We then sought to investigate whether the alterations in metabolite abundance were associated with changes in the expression of genes involved in methionine cycling. UNC1999-treated INA-6 cells downregulated genes involved in methionine metabolism such as methionine adenosyltransferase 2A (*MAT2A*), methionine adenosyltransferase 2B (*MAT2B*) and adenosyl-homocysteinase (*AHCY*). In addition, cystathionine-beta-synthase (*CBS*) and cystathionase (*CTH*) in the homocysteine degradation pathway decreased in expression post-treatment (Fig. 3e–i), in line with the observed accumulation of homocysteine (Fig. 3d). Consistent with a reduced glycine abundance and the accumulation of 5-methyltetrahydrofolic acid (Fig. 3a–b), UNC1999 treatment also decreased the expression of 5-methyltetrahydrofolate-homocysteinemethyltransferase (*MTR*), methylenetetrahydrofolate reductase (*MTHFR)* and serinehydroymethyltransferase 2 (*SHMT2*) (Fig. 3j–l). Finally, the expression of *MTAP* was also reduced in INA-6 cells after EZH2i (Fig. 3m), in line with the observed accumulation of 5-methylthioadenosine (Fig. 3c). A similar reduction in gene expression was observed in the other responsive cell lines (Supplementary Fig. 3o–w), while no decrease in expression of the above-mentioned genes was observed in the resistant cell line U1996 (Fig. 3e–m). To verify that the changes observed after UNC1999 were due to on-target effects, INA-6 and U1996 cells were treated with a different EZH2i, namely GSK343. A similar gene expression profile and viability effects were found to be induced as with UNC1999 (Supplementary Fig. 4a–l). Altogether, these data suggest that sensitivity to EZH2i was characterised by the downregulation of methionine cycling-associated genes.

### Methionine cycling genes were upregulated in MM patients

Our gene expression analysis suggested that EZH2i impaired the expression of genes involved in methionine cycling in sensitive cell lines. To investigate whether methionine cycling is altered in MM patients and, thus, whether targeting these genes would be of clinical relevance, we performed in silico analysis on patients’ gene expression data^22^ (Supplementary Fig. 5a–s). We found that *MAT2A, AHCY, MTR* and *MTAP* were increased in monoclonal gammopathy of undetermined significance (MGUS) and smouldering myeloma (SM) as compared to normal plasma cells^23^ (Supplementary Fig. 5a, c, f and h). Moreover, *MAT2A, MAT2B* and *MTR* were overexpressed in newly diagnosed MM patients as compared to MGUS patients (Supplementary Fig. 5i–j and n). Finally, the expression of *MAT2A*, *MAT2B* and *MTR* positively correlated with poor prognosis in patients not responding to bortezomib monotherapy^24^ (Supplementary Fig. 5q–s). In summary, methionine cycling-associated genes were found to be overexpressed in MM patients, pointing to them being of clinical relevance.

### Downregulation of methionine cycling genes by EZH2i was miRNA-dependent

**Fig. 4:**
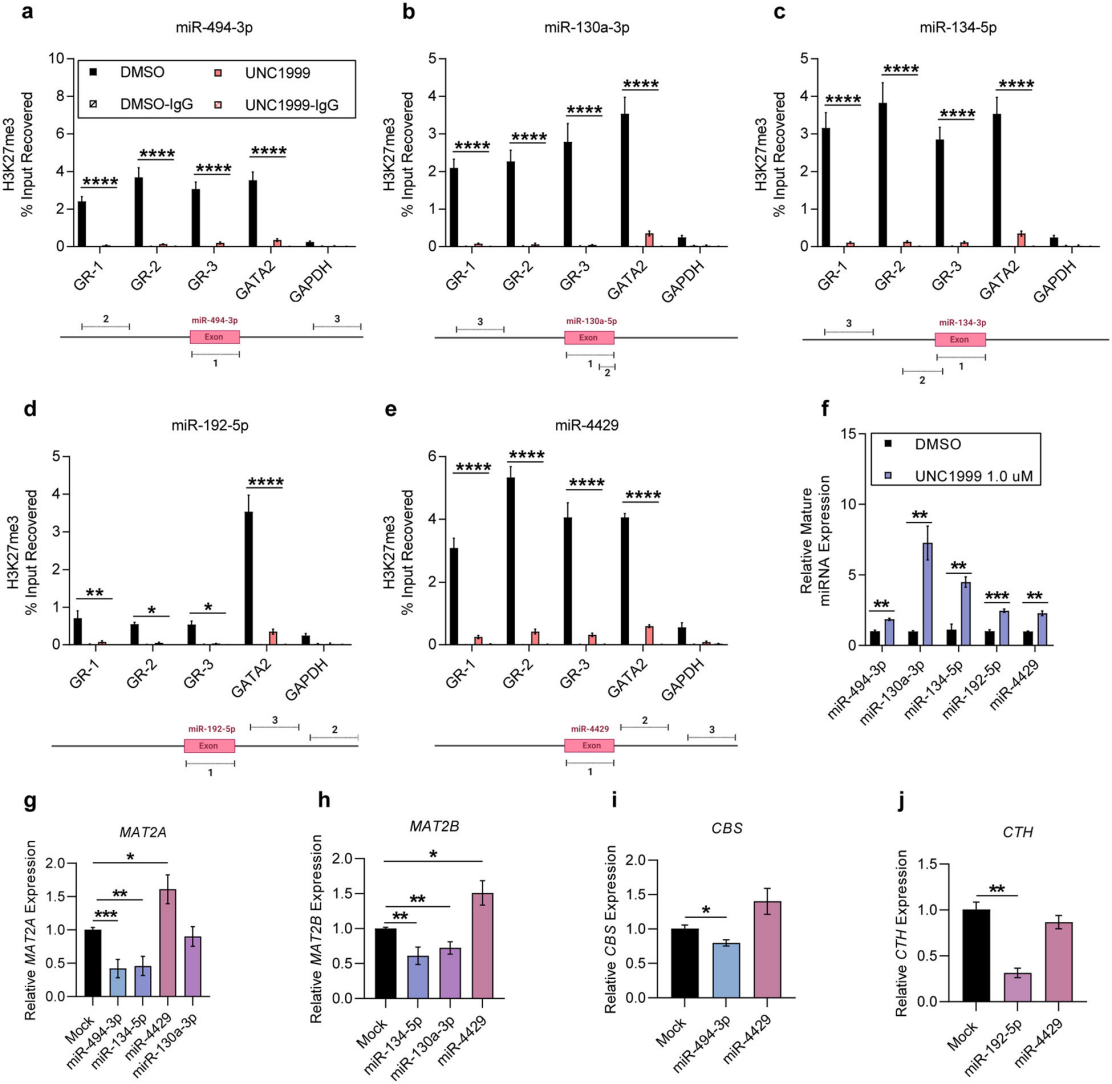
UNC1999 increased the expression of miRNAs that regulate methionine cycling-associated genes. **a**–**e** ChIP-qPCR analysis of H3K27me3 enrichment in UNC1999-treated INA-6 cells on exonic and gene body regions of **a** miR-494-3p, **b** miR-130a-3p, **c** miR-134-5p, **d** miR-192-5p and **e** miR-4429. For each miRNA, we analysed three genomic regions (GR-1, GR-2, GR-3). The location amplified by each primer pair is shown below every graph. Statistical analysis was performed with two-way ANOVA. GATA2 and GAPDH were used as positive and negative controls for H3K27me3 enrichment, respectively. The experiments were performed in three biological replicates. Values: mean with SEM. **f** RT-qPCR analysis of five PRC2-targeted miRNAs in INA-6 cells, post-UNC1999-treatment. Data were generated in three biological replicates. Value: mean with SEM. **g**–**j** MCF7 cells were transfected with 5 nM of miR-494-3p, miR-130a-3p, miR-134-5p, miR-4429 and miR-192-5p mimics. RT-qPCR analysis of methionine cycling-associated genes after 48 h of miRNA mimic treatment for **g** MAT2A, **h** MAT2B, **i** CBS and **j** CTH. miR-130a-5p or miR4429 were used as negative controls. Statistical analysis was performed with two-tailed *t*-test. Values: mean with SEM. Data were collected from three biological replicates. *p < 0.05, **p < 0.01, ***p < 0.001, ****p < 0.0001.

To investigate the molecular mechanisms underlying the downregulation of methionine cycling-associated genes in INA-6, we studied whether EZH2i induced expression of miRNAs that could regulate the genes of interest. In silico analysis using miRNA expression data from the INA-6 cell line^11^ identified 306 miRNAs that were upregulated upon UNC1999 treatment, 15 of which were predicted to target methionine cycling-associated genes according to an analysis performed using TargetScanHuman.org^25^ (Supplementary Fig. 6a–g). Of these, six miRNAs were significantly upregulated after UNC1999 treatment (i.e., miR-130a-3p, miR-134-5p, miR-192-5p, miR-4429, miR-223-3p and miR-320c) and four miRNAs showed a trend towards upregulation (i.e., miR-494-3p, miR-23a-3p, miR-21-5p and miR-27a-3p) (Supplementary Fig. 6h–v). However, only five of these miRNAs (i.e., miR-130a-3p, miR-134-5p, miR-192-5p, miR-4429 and miR-494-3p) were enriched for H3K27me3 under basal conditions (Supplementary Fig. 7a). These five also exhibited reduced H3K27me3 enrichment in three genomic regions post-UNC1999 treatment (Fig. 4a–e), which was associated with a significant increase in their relative expression (Fig. 4f). In addition, miR-494-3p, miR-130a-3p, miR-134-5p and miR-192-5p also showed reduced EZH2 binding after UNC1999 treatment (Supplementary Fig. 7b–d).

In order to investigate the functional relationship between each miRNA and its predicted target genes (Supplementary Figure 6g), we transfected MCF7 cells with the corresponding miRNA mimics (Supplementary Fig. 7g) and evaluated target gene expression. All five miRNA mimics were detectable 48-h post-transfection (Supplementary Fig. 7h–l). As predicted, *MAT2A* was downregulated upon overexpression of miR-494-3p or miR-134-5p, *MAT2B* expression was reduced upon transfection of miR-134-5p or miR-130a-3p, *CBS* was downregulated upon overexpression of miR-494-3p and *CTH* expression was reduced upon transfection with miR-192-5p (Fig. 4g–j). *MAT2A* levels, however, did not decrease upon overexpression of miR-4429 (Fig. 4g). Analysis in the U1996 cell line after UNC1999 treatment did not detect upregulation of any of the five miRNAs (Supplementary Fig. 7m).

Interestingly, DNA methylation was present in CpG sites surrounding miR-130a-3p and miR-4429 in U1996 cells, but not in INA-6 cells, suggesting that these miRNAs could be silenced by EZH2-independent epigenetic mechanisms in non-responsive cells (Supplementary Fig. 7n). Altogether, we identified five EZH2-targeted miRNAs, which were upregulated in INA-6 cells upon EZH2i and functionally regulated methionine cycling-associated genes.

### Sensitivity to EZH2i was associated with DNA damage, G2 arrest and induction of apoptosis

**Fig. 5:**
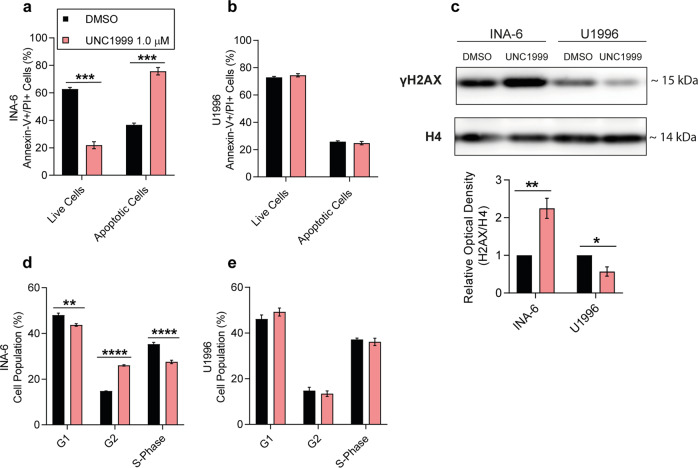
Sensitivity to EZH2 inhibition was associated with DNA damage, G2 arrest and apoptosis. **a**–**b** Flow cytometry analysis of apoptotic markers in (**a**) INA-6 and (**b**) U1996 cells upon UNC1999 treatment. **c** Representative western blot against γH2AX in INA-6 and U1996 cells and signal quantification of the western blot. Optical density was normalized against H4. The corresponding uncropped western blots can be found in Supplementary Fig. 9d. **d**–**e** Flow cytometry analysis of the cell cycle in (**d**) INA-6 and (**e**) U1996 cells upon UNC1999 treatment. Statistical analysis was performed with one-way ANOVA and multiple t-test. All experiments were performed in three biological replicates. Values: mean with SEM. *p < 0.05, **p < 0.01, ***p < 0.001, ****p < 0.0001.

Accumulation of homocysteine has been associated with an increase in reactive oxygen species (ROS), DNA damage and apoptosis in human leukemic cell lines and CBS-deficient leukemic patients^26,27^. Thus, we investigated whether such cytotoxic effects were induced upon UNC1999 treatment in INA-6 cells, which exhibited homocysteine accumulation as a result of treatment (Fig. 3d). Flow cytometry analysis revealed that the reduction in cell viability observed in INA-6 cells post-UNC1999 treatment was accompanied by induction of apoptosis (Fig. 5a and Supplementary Fig. 8a), while U1996 cells remained unaffected (Fig. 5b and Supplementary Fig. 8b). Moreover, enzymes involved in oxidative stress defence (i.e., SOD1 and TRX) were decreased in INA-6 after EZH2i treatment (Supplementary Fig. 8e–f), also in line with increased phosphorylation of histone H2AX (γH2AX) (Fig. 5c, d), a marker for DNA double-strand breaks. Interestingly, we observed opposite effects in U1996 after EZH2i, i.e., a trend towards increased protection from oxidative stress (Supplementary Fig. 8e–f) accompanied by a significant reduction of γH2AX (Fig. 5c), suggesting a functional DNA repair mechanism in the non-responsive cells. In addition, in INA-6 cells UNC1999 triggered the accumulation of cells in G2 phase of the cell cycle, coupled with a reduction of cells entering G1 and S-phase (Fig. 5d and Supplementary Fig. 8c), which was not observed for the U1996 cell line (Fig. 5e and Supplementary Fig. 8d). In summary, sensitivity to EZH2i was associated with accumulation of ROS, DNA damage, apoptosis and G2 arrest.

## Discussion

EZH2, the catalytic subunit of the PRC2 complex, is being extensively investigated as a potential target for cancer therapy and EZH2 inhibitors are in clinical trials for several cancer types^28^. We and others have previously demonstrated that PCR2-mediated gene silencing is a central mechanism for MM tumourigenesis^4,10^ which highlights EZH2 as a potential drug target for MM.

In this study, we evaluated the effect of EZH2i in vivo in the 5T33MM murine model using UNC1999, an EZH2 inhibitor that has shown a safe toxicity profile in mice^29^. EZH2i reduced the tumour burden, emphasising that targeting the PRC2 complex in MM could be of clinical relevance. However, the epigenetic landscape of MM patients is far more heterogeneous. In fact, we have previously investigated a cohort of patient-derived CD138^+^ MM cells and found that while UNC1999 induced a strong reduction in cell viability in most samples, several remained viable post-treatment^4^. Moreover, heterogeneous responses to EZH2i in human MM cell lines have been reported in studies using other EZH2 inhibitors^30,31^. Thus, it is of great importance to understand the mechanisms that underlie sensitivity to EZH2i in MM and to find signatures that can discriminate whether a patient is responding to EZH2i.

In diffuse large B-cell lymphoma, resistance to EZH2i can result froThus, we investigated whetherm EZH2 mutations that prevent successful binding of the inhibitor^32^. In MM, however, functional mutations of EZH2 have not yet been observed^5^. The activity of EZH2 depends on multiple metabolites and targeting of EZH2 affects the metabolism of cancer cells^33,34^. Thus, we investigated whether sensitivity and resistance to EZH2i could be discriminated using metabolic profiles. By performing LC-MS analysis, we found that the responsive cell line INA-6 and the non-responsive cell line U1996 shared a metabolic profile under basal conditions. Interestingly, however, they exhibited a differential metabolic response upon UNC1999 treatment. Specifically, UNC1999 triggered changes in metabolites involved in methionine cycling in responsive cells, such as accumulation of homocysteine, 5-methyltetrahydrofolic acid, 5-methylthioadenosine and decreased glycine abundance, which were not observed in non-responsive cells. These metabolic alterations may thus represent potential clinical biomarkers of sensitivity to EZH2i. Notably, methods for clinical quantification of 5-methyltetrahydrofolic acid and homocysteine levels in plasma are currently available^35,36^.

**Fig. 6:**
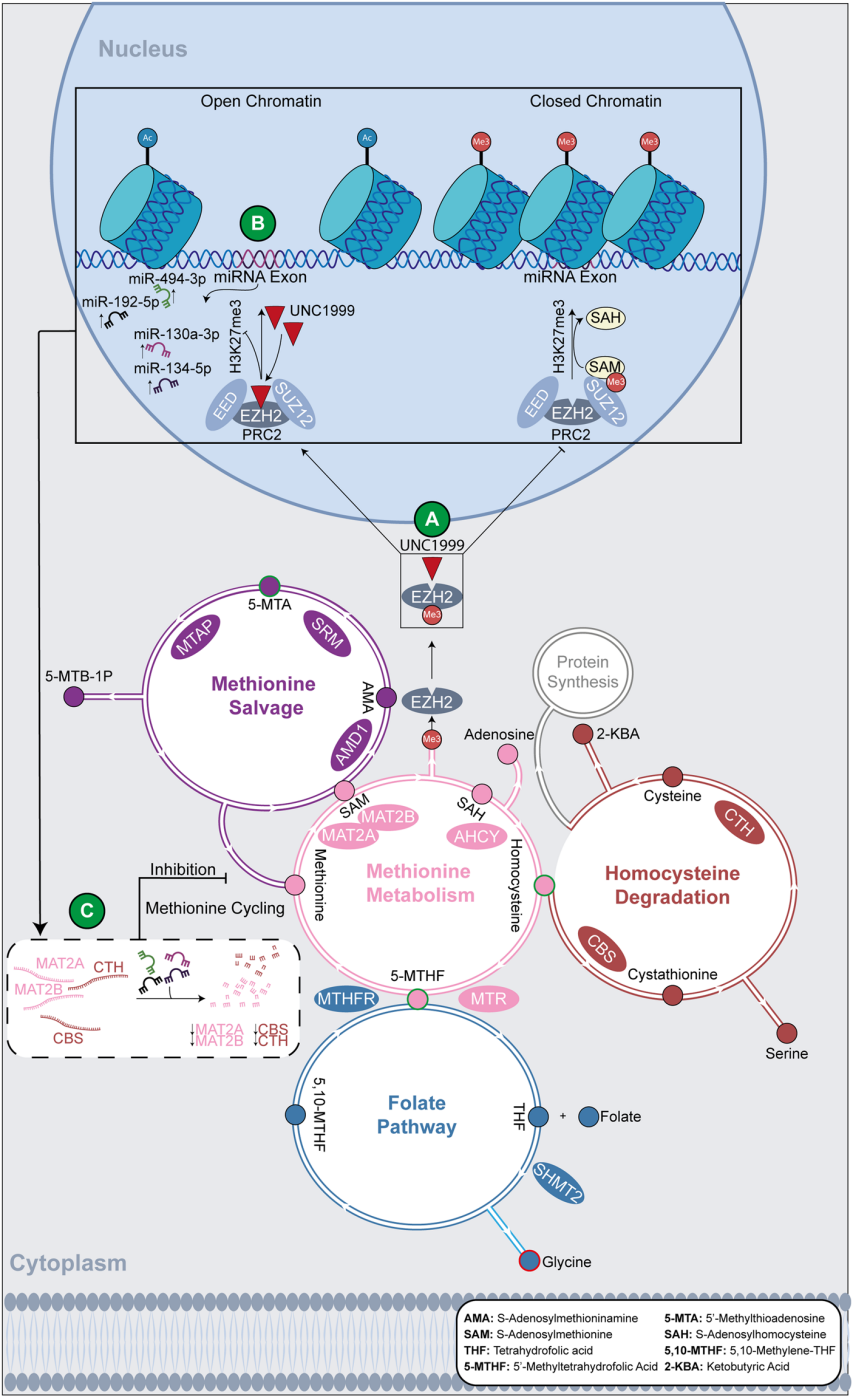
Schematic representation of the downstream effects of EZH2i on the metabolic profile of sensitive MM cell lines. **A** UNC1999 inhibits the activity of EZH2. **B** This prevents EZH2 from methylating H3, thus resulting in loss of H3K27me3 and open chromatin. As a consequence, the tumour-suppressor miRNAs miR-494-39, miR-192-5p, miR-130a-3p and miR-134-5p, which are silenced by H3K27me3 under basal conditions, are upregulated. **C** The tumour-suppressor miRNAs downregulate their target genes (*MAT2A*, *MAT2B*, *CBS* and *CTH*), which encode for enzymes involved in methionine cycling. This dysregulates methionine cycling and results in variation of metabolite abundance, such as accumulation of homocysteine. Metabolites in each pathway are indicated by small circles, which are colour coded with the same colour of the pathway they participate in. Metabolites that vary in abundance post UNC1999 treatment are marked by either a red (decreased abundance) or a green (accumulation) border.

As a next step, we sought to uncover the mechanisms underlying the metabolic alterations observed in cells sensitive to EZH2i. EZH2 activity is closely interconnected with the pathways involved in methionine cycling, such as methionine salvage, methionine metabolism, the folate pathway, and the homocysteine degradation pathway (Fig. 6). During methionine metabolism, the enzymes MAT2A/B convert L-methionine to SAM, which by donating a methyl group to EZH2, or other methyltransferases, is converted to SAH. In a second step, SAH is converted to homocysteine by the AHCY enzyme. Homocysteine can then be recycled back to methionine by MTR, which uses 5-methyltetrahydrofolic acid, (5-MTHF), as a methyl donor, or it can be degraded by CBS and CTH within the homocysteine degradation pathway^37,38^.

UNC1999 treatment of sensitive MM cells lines resulted in downregulation of multiple enzymes involved in methionine cycling (including *MAT2A*, *MAT2B*, *AHCY*, *MTR*, *CBS*, *CHT*, *MTHFR*, *SHMT2* and *MATP*), which is in line with a general shutdown of these pathways and likely underlies the observed metabolic changes. In fact, the downregulation of *CBS*, *CTH* and *MTR* suggests that homocysteine and 5-MTHF accumulation was due to impaired degradation and re-methylation of homocysteine to methionine. Downregulation of *MTHFR*, which converts 5,10-MTHF to 5-MTHF and of *SHMT2*, which converts THF into 5,10-MTHF generating the by-product glycine, are consistent with decreased glycine abundance. Finally, downregulation of *MATP* is consistent with the accumulation of 5-methylthioadenosine (Fig. 6).

Importantly, we found that some of the above-mentioned genes (i.e., *MAT2A*, *AHCY*, *MTR* and *MTAP*) were upregulated in MGUS and smouldering myeloma patients as compared to healthy individuals. Moreover, expression of *MAT2A*, *MAT2B* and *MTR* increased with disease progression and correlated with poor prognosis in bortezomib-resistant patients, suggesting that EZH2i could be beneficial for treatment for MM patients.

EZH2 mediates gene silencing by depositing H3K27me3, and its inhibition leads to increased expression of target genes. Thus, it was not directly clear how EZH2i resulted in the downregulation of methionine cycling-associated genes. Increased expression of miRNAs can downregulate target genes and was therefore a plausible mechanism. Consistent with our hypothesis, we found that UNC1999-treated cells upregulated four miRNAs (miR-494-3p, miR-130a-3p, miR-134-5p and miR-192-5p) that could functionally downregulate methionine cycling-associated genes such as *MAT2A*, *MAT2B*, *CBS* and *CTH*. Notably, these miRNAs have been reported in the context of several other cancer types^39–48^.

Finally, we investigated how this cascade of events was connected to cell death in sensitive cell lines. Literature shows that the trapping of homocysteine can induce accumulation of ROS, resulting in DNA damage and apoptosis in human leukemic cell lines and CBS-deficient patients^26,27^. Moreover, downregulation of *AHCY*, which is essential for maintaining cellular methylation^49^, has also been associated with DNA damage and cell cycle arrest^50^. In line with this, sensitive cells exhibited reduced oxidative stress defence, increased DNA double-strand breaks, G2 arrest and induction of apoptosis upon UNC1999 treatment.

In summary, we describe that sensitivity to EZH2i in MM is associated with a distinct metabolic signature, which results from a miRNA-mediated downregulation of genes involved in methionine cycling. Our study provides new insights into the metabolic response to targeted epigenetic treatment in MM and suggests that metabolic profiles have the potential as biomarkers for the response to EZH2i in MM.

## Materials and methods

### In vivo studies

C57BL/KaLwRij female mice were purchased from Envigo (Horst, The Netherlands). Mice were used at 6–10 weeks of age, housed, and treated following the conditions approved by the Ethical Committee for Animal Experiments, VUB (CEP 17-281-3). For evaluation of the effect of short-term UNC1999 treatment on H3K27me3 levels, naive mice were injected with 0.5 × 10^6^ 5T33MM cells at day 0. At day 14 post-inoculation, mice were randomly assigned to a treatment group receiving 150 or 300 mg/kg UNC1999^29^ daily for seven days (*n* = 6; oral gavage), or to a vehicle group receiving 10% N-methyl-2-pyrrolidinone in PEG300 daily for seven days (*n* = 6; oral gavage). We used a predefined value of *n* = 6 mice/treatment. Disease establishment was confirmed by analysis of bone marrow plasmacytosis quantified by May Grünwald-Giemsa staining of bone marrow cytospin samples. The animals were sacrificed when they showed signs of morbidity (paralysis) and the bone marrow harvested after flushing out of the femurs and tibiae and crushing out of the vertebrae. The bone marrow cells were suspended in serum-free medium (RPMI 1640; Cambrex, Europe), supplemented with penicillin-streptomycin, glutamine, and minimal essential medium (MEM) nonessential amino acid (NEAA)–pyruvate (Gibco, Life Technologies) and purified by Lympholyte M (Cedarlane, Hornby, ON, Canada) gradient centrifugation at 1000 × *g* for 20 min, generating enriched 5T33MM cells. For further enrichment, CD11b+ cells were depleted using a MidiMACS magnetic cell separator, LD separation columns and CD11b magnetic microbeads (all from Miltenyi Biotec, Bergisch Gladbach, Germany). 5T33MMvv cells at a concentration of 1 × 10^7^ cells per 90 µl MACS buffer (phosphate-buffered saline containing 0.5% bovine serum albumin and 2 nM EDTA, pH 7.2) and 10 µl CD11b Microbeads were incubated at 4 °C for 15 min. The 5T33MMvv-microbeads mixture was then loaded onto a separation column placed on the magnetic cell separator and the flow through cell fraction was collected for downstream experiments.

To evaluate the effect of UNC1999 on tumour burden in vivo, 5T33MM cells were injected in C57BL/KalwRij mice as described above. At day 2, mice were randomly assigned to a treatment group receiving 200 mg/kg UNC1999 every other day (*n* = 10; oral gavage), or to a vehicle group receiving 10% N-methyl-2-pyrrolidinone in PEG300 every other day (*n* = 10; oral gavage). We performed Wilcoxon–Mann–Whitney power analysis by using GPower^51^ 3.1 with a power of 0.9 and an effect size of 2. Further considering a drop-out rate of ~10%, resulted in 10 mice/group. The animals were sacrificed when they showed signs of morbidity (paralysis). Tumour load was analysed by means of serum paraprotein concentration quantified by electrophoresis and assessment of total protein; bone marrow plasmacytosis quantified by May Grünwald-Giemsa staining of bone marrow cytospin samples; and spleen weight.

Mice that did not develop the disease or died for other reasons than disease progression were excluded from the analysis.

### Cell culture

The MM cell lines U1996, U266-1970, KMS-28PE, OPM2, L363, LP-1, INA-6 and JJN3 were cultured in RPMI-1640 AQmediaTM media (Gibco; Thermo Fisher Scientific, Inc., Waltham, MA, USA; cat. no 31870025) with 10% foetal bovine serum (FBS; Gibco; cat. no 10270106), 1% GlutaMAX^™^-I X100 (Gibco; cat. no 35050038) and antibiotics (streptomycin 100 µg/mL and penicillin 100 U/mL; Gibco; cat. no 15140122)^52^. INA-6, U266-1970 and U1996 were supplemented with 10 ng/mL IL-6 (Prepotech; Cranbury, NJ, USA; cat. no 200-06).

MCF7 cells were cultured in Dulbecco’s Modified Eagle Medium/Nutrient Mixture F-12 (DMEM/F-12; Gibco; cat. no 11320033) with 10% FBS (Gibco), 1% GlutaMAX^™^-I X100 (Gibco) and antibiotics (streptomycin 100 µg/mL and penicillin 100 U/mL; Gibco). All cell lines were cultured at 37 °C in a humidified 5% CO_2_ in-air atmosphere.

For all cell lines, and prior to experiments, we confirmed the absence of Mycoplasma infection by using MycoAlert™ Mycoplasma Detection Kit (Lonza; Basel, Switzerland; cat. no LT07118) and measurement of luminescence on Lumat LB9597 (Berthold; Bad Wildbad, Germany).

### UNC1999 treatment of MM cell lines

Seven authenticated MM cell lines were selected for UNC1999 drug response analysis^29^. Initially, 100,000 cells/mL were seeded in flasks 24 h prior to the addition of 1 µM UNC1999 or DMSO (Sigma-Aldrich; Merck; Darmstadt, Germany; cat. no 317275). The cells received media and reagents change after 3 days. All experiments were performed after 5 days of treatment in cells originating from 3 independent cell batches per cell line.

### GSK343 treatment of MM cell lines

INA-6 and U1996 MM cell lines were selected for drug response analysis to the alternate EZH2 inhibitor GSK343 (Tocris; Bio-Techne; Minneapolis, MN, USA; cat. no 6128). Initially, 100,000 cells/mL were seeded in flasks for 24 h prior to 0.5 µM GSK343 or DMSO (Sigma-Aldrich) treatment. The cells received media and reagents change after 3 days. Cells were harvested after 5 days. All experiments were performed in three independent cell batches per cell line.

### LC-MS chemicals

Ammonium formate (LC-MS grade), formic acid (LC-MS grade) and methanol (LC-MS grade) were purchased from Sigma-Aldrich; acetonitrile (LC-MS grade) from Fisher Scientific (Zurich, Switzerland) and chloroform (analytical grade) from BDH Laboratory Supplies (Poole, England, UK). The water used was purified using a Milli-Q^TM^ water system from Millipore (Bedford: MA, USA).

### LC-MS sample preparation/metabolite extraction

For LC-MS, cells were washed in ice-cold PBS and snap frozen. Cell lysis was conducted by two steps of 10 min of thawing-freezing cycles at 37 °C or in liquid N_2_, respectively. After, the samples were sonicated at ultrasonic wave output power 320 W in Bioruptor® (Diagenode; Liège, Belgium) for 30 s and centrifuged (2000 RCF, 10 min, 4 °C). The supernatants were transferred to fresh extraction tubes, and a QC sample was prepared by pooling an equal volume from each sample into a separate extraction tube. Methanol and chloroform were added to the tubes to a final proportion of 2.85:4:4 (water:methanol:choloroform), the tubes gently vortexed and incubated at 6 °C for 20 min. After centrifugation (2000 RCF, 20 min, 4 °C), the supernatants were transferred to fresh extraction tubes and evaporated at 40°C under N_2_(g). The samples were then reconstituted in acetonitrile:water 75:25^53–55^ *n*(INA-6 DMSO) = 9, *n*(INA-6 UNC1999) = 9; *n*(U1996 DMSO) = 11, *n*(U1996 UNC1999) = 10; *n*(KMS-28PE DMSO) = 4, *n*(KMS-28PE UNC1999) = 4; *n*(U266-1970 DMSO) = 4, *n*(U266-1970 UNC1999), *n*(LP-1 DMSO) = 4, *n*(LP-1 UNC1999) = 4; *n*(L363 DMSO) = 4, *n*(L363 UNC1999) = 4; *n*(OPM2 DMSO) = 4, *n*(OPM2 UNC1999) = 4.

### LC-MS analysis and metabolic profiling

Reconstituted samples were analysed using an Acquity I-Class UPLC equipped with a BEH Amide column (2.1 × 50 mm, 1.7 µm i.d.) hyphenated to a Synapt G2S qTOF through an electrospray ionization (ESI) source, all from Waters (Waters Corporation; Saint-Quentin En Yvelines Cedex, France). MassLynx v. 4.1 (Waters Corporation) was used for instrument control and data collection.

The chromatographic separation was performed at 40 °C and using 5 µL injection volumes. Mobile phase A consisted of 95:5 acetonitrile:water, with 10 mM ammonium formate and 0.1% formic acid, while mobile phase B consisted of 50:50 acetonitrile:water, with 10 mM ammonium formate and 0.1% formic acid. The flow rate was set to 0.3 mL/min, and the gradient started with 100% for 0.5 min, followed by a non-linear gradient (MassLynx slope factor 8) over 12.5 min to 100% B, then isocratic at 100% B for 4 min, and finally 100% A for 6 min to re-equilibrate the column, i.e., a total runtime of 23 min per injected sample.

Detection was performed in resolution MS^E^ mode, in the range *m/z* 50–800. All samples were analysed in positive mode first, followed by negative mode. The capillary voltage was 1 and −2 kV and cone voltage 30 and 25 V for positive and negative mode, respectively. The source temperature was 120 °C and the de-solvation temperature 500 °C in both modes. Nitrogen was used as de-solvation and cone gas, at a flow of 800 and 50 L/h, respectively, in both modes. For MS^E^ acquisition, a collision energy ramp from 20 to 45 eV was used, with argon as the collision gas. Lock-mass correction was applied using a solution of leucine-enkephalin.

The QC sample (see sample extraction section) was injected repeatedly before sample analysis to condition the system, to ensure stable analytical conditions, including mass accuracy, instrument sensitivity and chromatographic performance. Furthermore, the QC sample was also injected at regular intervals (every 6th injection) to monitor analytical stability throughout the analysis^56^.

### LC-MS data processing

Data quality was assessed using univariate data analysis of a selected set of metabolites, comprising mass accuracy, retention time and peak area of the QC samples throughout the entire analysis. Raw data files were then converted to NetCDF file format using Databridge (Masslynx version 4.1; Waters S.A.S.). The R-based XCMS package was used for peak detection, retention time alignment and peak grouping^57^.

Briefly, the “centWave” function was used for peak detection in the 0.75–17 min retention time range, with 10 ppm maximum *m/z* deviation between scans, 5–45 s peak width boundary and signal-to-noise ratio cut-off at five. The “obiwarp” function was used for retention time alignment. For adduct and isotope annotation of the data, R-based package CAMERA was used^58^. The resulting dataset was exported to Microsoft Excel for probabilistic quotient normalization (PQN)^59^. After normalization, all features with a coefficient of normalization (CV) > 30% in the QC injections were removed^56,60,61^.

### LC-MS multivariate and univariate data analysis

The normalized dataset was imported to Simca P + (version 15, Sartorius Stedim Data Analytics AB, Umeå, Sweden) and pareto scaled. PCA was used to analyse sample groupings, detect outliers and search for systematic trends in the data. Orthogonal projections to latent structures—discriminant analysis (OPLS-DA) was used in combination with shared and unique structures (SUS) plots to pinpoint features related to differences between cell lines as well as treated and control samples^62,63^. OPLS-DA models were built for each set of samples from different cell lines, with treated/control as the discriminating factor. Separate models were built for responsive (INA-6) and non-responsive (U1996) cell lines, respectively. The latter two models were offset using a SUS-plot, and features with *p*(corr) ≥ 0.4 were annotated.

For identification, molecular weight, isotopic patterns, fragmentation and, when possible, retention time comparison with an in-house database was used. The Human Metabolome Database (HMDB) and METLIN were used for experimental *m/z* search with a maximum molecular weight difference of 30 ppm. All annotated metabolites should be considered putatively annotated (level 2) according to the Metabolomics Standards Initiative nomenclature^64^.

Annotated metabolites were subjected to Pathway analysis using MetaboAnalyst 4.0^21^. Metaboanalyst was also utilized for heatmap generation of the whole dataset, as well as annotated subsets. One-way analysis of variance (ANOVA) with post hoc Tukey tests were used for significance testing of metabolites, and *p*-values < 0.05 were considered significant.

### RNA extraction, cDNA synthesis and Real-Time quantitative PCR

RNA was extracted using TRIzol™ Reagent (Invitrogen; Thermo Fisher Scientific, Inc., cat. no15596018) as described by the manufacturer. Conversion to cDNA was performed by using the Superscript III Reverse Kit Transcriptase (Invitrogen, CA, USA; cat. no 18080044) utilizing 1 µg of RNA template. Real-time quantitative PCR analysis was performed with SSOAdvanced Universal SYBR® Green Supermix (Bio-Rad Laboratories, Inc.; CA, USA; cat. no 1725271) using 0.25 µM of forward and reverse primers found in Supplementary Table II in three replicates. miRNA reverse transcription was performed using 10 ng of RNA and by utilizing TaqMan® MicroRNA Reverse Transcription Kit (Applied Biosystems; Thermo Fisher Scientific, Inc., cat. no 4366596). TaqMan® gene expression assays were used for miR-494-3p, miR-130a-3p, miR-134-5p, miR-192-5p and miR-4429, in 3 technical replicates. CFX96 Touch Real-Time PCR Detection System (Bio-Rad Laboratories, Inc.) was used for fluorophore detection. Relative gene and miRNA expression analysis was performed in Bio-Rad CFX Maestro V.1.1 (Bio-Rad Laboratories, Inc.) and calculated with 2^−ΔΔCT^ with actin or RNU6B as a reference gene for protein-coding genes and miRNA expression, respectively. All samples were analysed in biological and technical triplicates.

### Protein extraction, western blot and image analysis

MM cell lines, MCF7 cells and 5T33MM bone marrow cells were harvested and washed in ice-cold PBS (Gibco; cat. no 70011044) and collected post centrifugation at 100 RCF for 5 min. Histone proteins were extracted by utilizing a histone extraction kit (Abcam plc.; Cambridge, UK; cat. no 113476) following the manufacturer’s instructions. Protein extraction was conducted using RIPA extraction buffer (Millipore; Merck; cat. no 20188) with protease inhibitors (cOmplete™, EDTA-free Protease Inhibitor Cocktail; Roche; Merck; cat. no 11873580001). Western blot was performed as previously described^4^, using anti-H3K27me3 (1:1000) (Active Motif; Waterloo, Belgium; cat. no 39155), anti-γH2AX (1:500) (Millipore; Darmstadt, Germany; cat. no 05-636-I), oxidative stress defence cocktail (1:250) (Abcam; cat. no ab179843) and total histone H3 (1:3000) (Abcam; Cambridge, UK; cat. no 1791) antibodies. Total histone H4 (1:1000) (Active Motif; Waterloo, Belgium; cat. no 91295) and actin (1:500) (Santa Cruz Biotechnology, Inc.; Dallas, TX, USA; cat. no sc-47778) were used as loading controls.

Western blot image analysis and quantification was performed in ImageJ V.1.52a^65^ and normalized against total histone H4. Cell line western blot quantifications were analysed by multiple t-test correcting for multiple testing by the Holm-Sidak method.

### Chromatin immunoprecipitation

ChIP was performed utilizing the iDeal ChIP-seq kit for histones (Diagenode; Seraing, Belgium; cat. no C01010051) according to the manufacturer’s protocol. Chromatin from 7 million cells was crosslinked for 8 min in 1% formaldehyde (Thermo Scientific; Thermo Fisher Scientific, Inc., cat. no 28906) at RT, followed by the addition of 0.1 M of glycine to inhibit the crosslinking. The chromatin was sonicated for 10 cycles on Pico Bioruptor™ (Diagenode) (30 s ON/30 s OFF). For each immunoprecipitation reaction, the chromatin of 200,000 cells was combined with either 5 µg anti-H3K27me3 (Active Motif; cat. no 39155), 5 µg anti-IgG Rabbit (Millipore, cat. no PP64B), 1 µg anti-EZH2 (Millipore, cat. no 17-662) or 1 µg anti-IgG Mouse (Millipore; CS200621) antibodies. 10% of the chromatin used for the IPs was de-crosslinked and purified prior to immunoprecipitation and used as input. qPCR analysis was conducted by SSOAdvanced Universal SYBR® Green Supermix (Bio-Rad Laboratories, Inc.; CA, USA, cat. no 1725271), utilizing 0.25 µM of forward and reverse primers found in Supplementary Table II, in two technical replicates. CFX96 Touch Real-Time PCR Detection System (Bio-Rad Laboratories, Inc.) was used for fluorophore detection. Data analysis was performed using % input = 2^[(Ct_input_ − log2^10^ − Ct_sample_] × 100, as specified by the manufacturer’s protocol and plotted against the positive control GATA2 and negative control GAPDH^7^.

### Cell viability assay

Resazurin reduction protocol was used to evaluate cell viability after UNC1999 treatment^66^. Cells were seeded into a 96-well plate and alamarBlue™ solution (Sigma-Aldrich, MI, USA, cat. no R7017) was added at 10% v/v in 3 technical replicates. The fluorescent signal was measured by Synergy HTX Plate Reader (BioTek; Winooski, VT, USA) at a threshold of five times the background.

### Cell viability staining by flow cytometry

Cells were stained using the Zombie Aqua™ Fixable Viability Kit (BioLegend; San Diego, CA, USA; cat. no 423101) following the manufacturer’s instructions. The samples were analysed on the CytoFLEX LX (Beckman Coulter; Brea, CA, USA). Data were analysed using CytExpert V.2.4.0.28 (Beckman Coulter).

### Apoptosis and cell cycle analysis

Apoptosis assay was performed by harvesting the cells by 210 RCF centrifugation for 5 min. The samples were then washed with PBS and centrifuged for an additional 5 min. The samples were then re-suspended in FITC-conjugated Annexin V and propidium iodine using the TACS Annexin V-FITC Apoptosis Kit (R&D Systems, Gaithersburg, MD, USA). Cell cycle assay was performed as per the manufacturer’s instructions, utilizing BrdU incorporation for 1 h of sample incubation (559619, BD Pharmingen, CA, USA). All data were collected on the BD LSR Fortessa flow cytometer (BD Bioscience, CA, USA). Data were analysed with Kaluza Analysis flow cytometry software V.1.3 (Beckman Coulter, Brea; CA, USA) and Graphpad Prism V.8.4.3 (Graphpad Software Inc, CA, USA).

### MiRNA mimics

MCF7 cells were seeded at concentration 120,000/well in 6-well plates and allowed to rest for 24 h prior to transfection. Transfections were performed in at least three biological replicates using RNAiMAX lipofectamine (Invitrogen; cat. no 13778075) according to the manufacturer’s instructions and 5 nM of miCURY LNA miRNA mimics (QIAGEN; Supplementary Table IV). Transfection efficiency was evaluated at 24-h post-transfection using 5′-FAM-labbeled negative control mimic on CytoFLEX LX (Beckman Coulter, Brea; CA, USA). Data were analysed using CytExpert V.2.4.0.28 (Beckman Coulter). Detection of mimics and target genes by RT-qPCR was evaluated at 48-h post-transfection.

### In silico analysis

Normalized (MAS5) patient gene expression and survival data (U133 Plus 2.0; Affymetrix, CA, USA) (GEO:GSE2113, GSE9782 and GSE5900)^22–24,67^ were obtained through genomicscape.com. INA-6 miRNA array expression data was obtained from GSE:87715^11^. All data were analysed in Graphpad Prism V.8.4.3 (Graphpad Software Inc, CA, USA). miRNA target prediction was determined by TargetScanHuman^25^.

### DNA methylation array

DNA was extracted from three independent cell batches of the U1996 and the INA-6 MM cell lines using the PureLink^®^ Genomic DNA Mini Kit (Invitrogen, CA, USA) as per manufacturer’s protocol. Bisulfite conversion was performed using the EZ DNA Methylation™ Kit (#D5004, Zymo Research, CA, USA) with 250 ng of DNA per sample. The bisulfite-converted DNA was eluted in 15 μl according to the manufacturer’s protocol, evaporated to a volume of <4 μl, and used for methylation analysis by the Illumina Infinium EPIC array^68^^,69^.

### Statistical analysis

Student *t*-test, multiple *t*-test and one-way ANOVA were utilized to analyse all mice experiments, MGUS-MM patient gene expression data and metabolite statistics, respectively. miRNA array data were analysed by student *t*-test. All statistical analyses for apoptosis, cell cycle and ChIP-qPCR were done by two-way ANOVA. Multiple *t*-test was used to analyse RT-qPCR (no correction for multiple testing) and one-way ANOVA was used to analyse normal PC, MGUS and SM patient gene expression and cell viability data. All data were processed in Graphpad Prism V.8.4.3 (Graphpad Software Inc, CA, USA).

## Supplementary information

Supplementary Tables and Figures

## Data Availability

The accession number for DNA methylation array data reported in this paper is GEO:GSE152006.
