# Supplementary material for: A distinct metabolic response characterizes sensitivity to EZH2 inhibition in multiple myeloma
Source: Cell Death Dis. 2021 Feb 12;12(2):167. doi: 10.1038/s41419-021-03447-8 (PMC7881125; doi:10.1038/s41419-021-03447-8)
Supplement: Supplementary file 1 — Supplementary Tables and Figures [file 41419_2021_3447_MOESM1_ESM.pdf]

A distinct metabolic response characterizes sensitivity to EZH2 inhibition in multiple myeloma

Patrick Nylund, Alba Atienza Párraga, Jakob Haglöf, Elke De Bruyne, Eline Menu, Berta Garrido-Zabala, Anqi Ma, Jian Jin, Fredrik Öberg, Karin Vanderkerken, Antonia Kalushkova and Helena Jernberg-Wiklund.

Supplementary Information

Tables

Supplementary Table I. Changes in amino acid synthesis upon UNC1999 treatment.

	INA-6		U1996	
**Amino acids**	**Fold Change**	**P-Value (<0.05)**	**Fold Change**	**P-Value (<0.05)**
(iso)Leucine	0,68	/	0,89	/
Alanine	1,06	/	0,87	/
Arginine	0,87	/	0,96	/
Cysteine	0,85	/	1,25	/
Glutamic Acid	0,90	/	1,02	/
Glycine	0,64	0,01	0,99	/
Lysine	0,89	/	1,09	/
Methionine	0,90	/	1,09	/
Phenylalanine	0,64	/	0,99	/
Proline	0,43	0,04	0,99	/
Serine	1,51	/	0,91	/
Tryptophan	1,15	/	1,23	/
Tyrosine	0,69	0,04	0,82	/
Valine	0,63	0,02	0,90	0,05

Supplementary Table II. Primers used for gene/miRNA relative expression analysis by qPCR.

Gene	Forward Primer	Reverse Primer
AHCY	ATCCTTGGCCGGCACTTTGAG	TCCACCTGCGGCTTGATGTTC
MTAP	TTGCCCCAAAACGAGAGAGG	CTTTCTGCCCGGGAGCTAAA
SHMT2	GCTGCCCTAGACCAGAGTTG	GAGCCGCCCAAAACAAAGAG
MTR	CTGAAGAAAACCCTGCGGGA	TCCCTCCATCCAGCACCATA
MTHFR	CCGCCGTGAACTACTGTGG	AGATGGCCCGTGATCTCCTC
CBS	ACATGCTCTCGTCCCTGCTT	GTGAGGCGGATCTGTTTGAACT
CTH	CATGAGTTGGTGAAGCGTCAG	AGCTCTCGGCCAGAGTAAATA
MAT2A	ACCAGAAAGTGGTTCGTGAAG	CCAGGCTACCAGCACGTTACA
MAT2B	TTCACTGGTCTGGCAATGAAC	AGGGCTGTCAGTAATAGGTCTT
ACTIN	CCAACCGCGAGAAGATGA	TCCATCACGTGCCAGTG
**ChIP-miRNA**	**Forward Primer**	**Reverse Primer**
miR-130a Genomic Region 1	TGTGCTACTGTCTGCACCTG	ACACGGCCAATGCCCTTTTA
miR-130a Genomic Region 2	TGTGCTACTGTCTGCACCTG	ACACGGCCAATGCCCTTTTA
miR-130a Genomic Region 3	TGACCTCCAGACTTTGGGGA	ACAGGACCCACTAAAGCAGC
miR-192 Genomic Region 1	CAGCCAGTGCTCTCGTCTC	AGGCGAACATACCTGTGACC
miR-192 Genomic Region 2	AAGCCTCGGTGAAAAGACCA	GAACCAATTGGAGACCGGGG
miR-192 Genomic Region 3	AGAGGTGACAGTGGCCAAAC	GGAGCGGCTCGGATTTACAA
miR-494 Genomic Region 1	CTCGAAGGAGAGGTTGTCCG	GAGGTTTCCCGTGTATGTTTCA
miR-494 Genomic Region 2	AGAGGGCAGAGAGAGTGTGT	TGTCCTTTGACCCCTCCGTA
miR-494 Genomic Region 3	GCTCTCTTGACGCACTGACT	GAGCTTTCCTGACGGTGGAA
miR-4429 Genomic Region 1	ATTACTGGAGCACCCCATTCC	GCCAACACAGAAGCATGGTGA
miR-4429 Genomic Region 2	GGCTGGGGAATTCTTCCAGTTA	CAGACATCACTGACCCATGC
miR-4429 Genomic Region 3	CCAGGCAGTCTGAGTTGGAG	TGAGAGGCGACTGGTGTCTA
GATA2	ATCAGTGAGTGCGTGTGCTC	ATCAGTGAGTGCGTGTGCTC
GAPDH	TACTAGCGGTTTTACGGGCG	TCGAACAGGAGGAGCAGAGAGCGA

Supplementary Table III. Sequences of miRNA mimics.

miRNA Mimics	miRNA Strand	Company
LNA miRNA miRCURY Negative Control	FAM-5’GAUGGCAUUCGAUCAGUUCUA	QIAGEN
LNA miRNA-494-3p Mimic	5'UGAAACAUACACGGGAAACCUC	QIAGEN
LNA miRNA-130a-3p Mimic	5'CAGUGCAAUGUUAAAAGGGCAU	QIAGEN
LNA miRNA-134-5p Mimic	5'UGUGACUGGUUGACCAGAGGGG	QIAGEN
LNA miRNA-4429 Mimic	5'AAAAGCUGGGCUGAGAGGCG	QIAGEN
LNA miRNA-192-5p Mimic	5'CUGACCUAUGAAUUGACAGCC	QIAGEN

Supplementary Figures

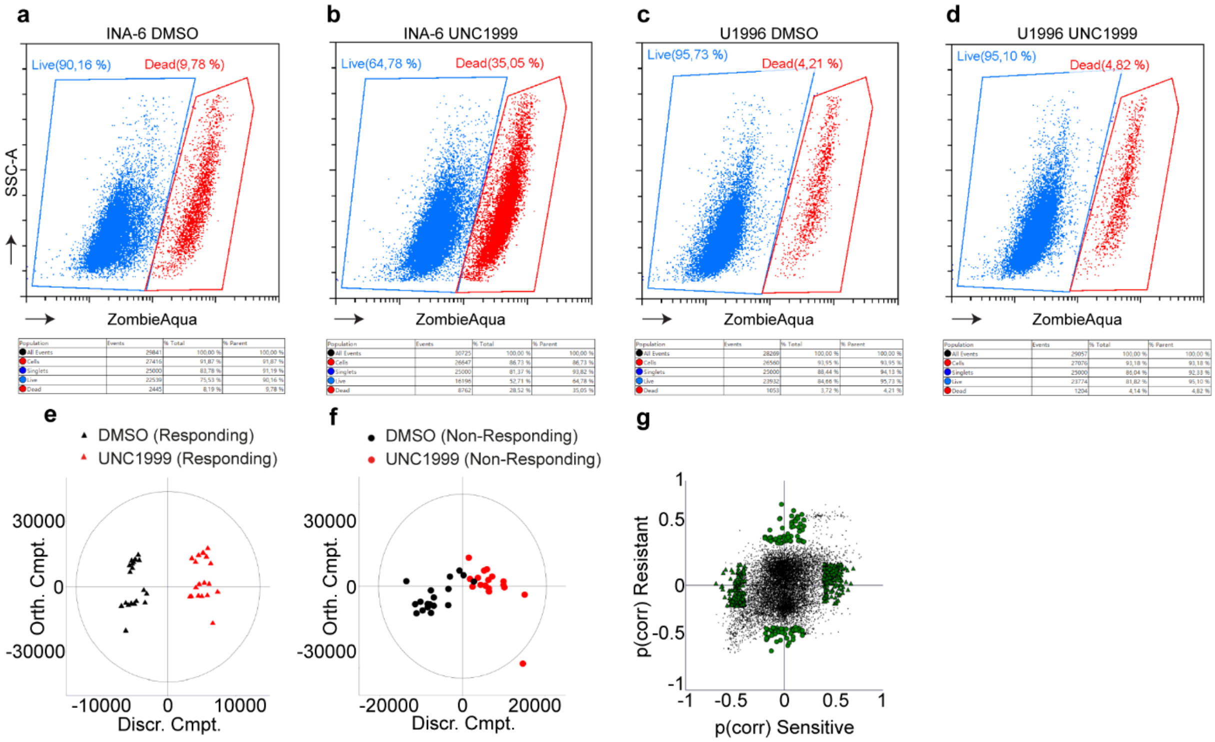

Supplementary Figure 1. Treatment of human MM cell lines with UNC1999 yielded differential viability and metabolic response. a-d) Representative FACS plots of live-dead staining of (a-b) INA-6 cells and (c-d) U1996 cells treated with DMSO or UNC1999. Each panel is representative of three biological replicates. **e)** OPLS-DA plot of dysregulation of metabolites in all responsive cell lines. n = 21 replicates per group (9 replicates of INA-6, 4 replicates of LP-1, L363 and OPM2). **f)** OPLS-DA plot of dysregulation of metabolites in all non-responsive cell lines. n_{DMSO} = 19 replicates (11 replicates of U1996, 4 replicates of KMS-28PE and U266-1790); $n_{UNC1999}$ = 18 replicates (10 replicates of U1996, 4 replicates of KMS-28PE and U266-1790). **g)** SUS-plot of discriminating metabolites used for metabolite selection. The Y- and X-axis display discriminating magnitude, while the transverse detection describes commonly shared metabolites between responsive and non-responsive samples. The metabolites with a greater discriminating magnitude are shown in green.

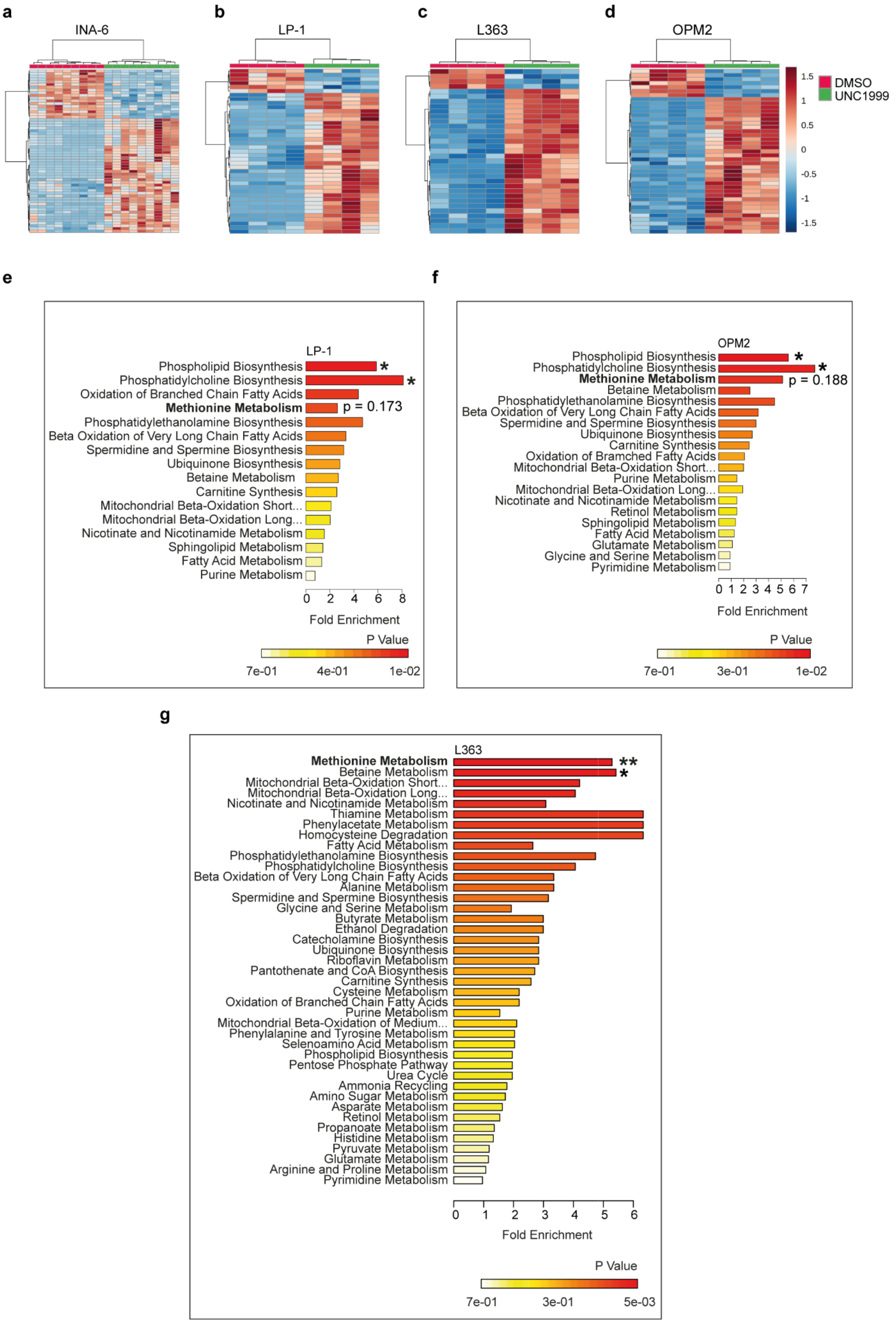
a
INA-6
b
LP-1
c
L363
d
OPM2
1.5
1
0.5
0
-0.5
-1
-1.5
DMSO
UNC1999
e
LP-1
Phospholipid Biosynthesis *
Phosphatidylcholine Biosynthesis *
Oxidation of Branched Chain Fatty Acids
Methionine Metabolism p = 0.173
Phosphatidylethanolamine Biosynthesis
Beta Oxidation of Very Long Chain Fatty Acids
Spermidine and Spermine Biosynthesis
Ubiquinone Biosynthesis
Betaine Metabolism
Carnitine Synthesis
Mitochondrial Beta-Oxidation Short...
Mitochondrial Beta-Oxidation Long...
Nicotinate and Nicotinamide Metabolism
Sphingolipid Metabolism
Fatty Acid Metabolism
Purine Metabolism
0 2 4 6 8
Fold Enrichment
P Value
7e-01 4e-01 1e-02
f
OPM2
Phospholipid Biosynthesis *
Phosphatidylcholine Biosynthesis *
Methionine Metabolism p = 0.188
Betaine Metabolism
Phosphatidylethanolamine Biosynthesis
Beta Oxidation of Very Long Chain Fatty Acids
Spermidine and Spermine Biosynthesis
Ubiquinone Biosynthesis
Carnitine Synthesis
Oxidation of Bramched Fatty Acids
Mitochondrial Beta-Oxidation Short...
Purine Metabolism
Mitochondrial Beta-Oxidation Long...
Nicotinate and Nicotinamide Metabolism
Retinol Metabolism
Sphingolipid Metabolism
Fatty Acid Metabolism
Glutamate Metabolism
Glycine and Serine Metabolism
Pyrimidine Metabolism
0 1 2 3 4 5 6 7
Fold Enrichment
P Value
7e-01 3e-01 1e-02
g
L363
Methionine Metabolism **
Betaine Metabolism *
Mitochondrial Beta-Oxidation Short...
Mitochondrial Beta-Oxidation Long...
Nicotinate and Nicotinamide Metabolism
Thiamine Metabolism
Phenylacetate Metabolism
Homocysteine Degradation
Fatty Acid Metabolism
Phosphatidylethanolamine Biosynthesis
Phosphatidylcholine Biosynthesis
Beta Oxidation of Very Long Chain Fatty Acids
Alanine Metabolism
Spermidine and Spermine Biosynthesis
Glycine and Serine Metabolism
Butyrate Metabolism
Ethanol Degradation
Catecholamine Biosynthesis
Ubiquinone Biosynthesis
Riboflavin Metabolism
Pantothenate and CoA Biosynthesis
Carnitine Synthesis
Cysteine Metabolism
Oxidation of Branched Chain Fatty Acids
Purine Metabolism
Mitochondrial Beta-Oxidation of Medium...
Phenylalanine and Tyrosine Metabolism
Selenoamino Acid Metabolism
Phospholipid Biosynthesis
Pentose Phosphate Pathway
Urea Cycle
Ammonia Recycling
Amino Sugar Metabolism
Asparate Metabolism
Retinol Metabolism
Propanoate Metabolism
Histidine Metabolism
Pyruvate Metabolism
Glutamate Metabolism
Arginine and Proline Metabolism
Pyrimidine Metabolism
0 1 2 3 4 5 6
Fold Enrichment
P Value
7e-01 3e-01 5e-03

Supplementary Figure 2. MM cell lines responding to UNC1999 treatment share the common feature of methionine cycling dysregulation. a-d) Heatmaps based on the top discriminating metabolites between DMSO and UNC1999 treatment for a) INA-6 (n = 9 per group), b) LP-1(n = 4 per group), c) L363 (n = 4 per group) and d) OPM2 (n = 4 per group). **e-g)** Metabolite set enrichment analysis (MSEA) based on the discriminating metabolites in (e) LP-1, (f) OPM2 and (g) L363 post UNC1999 treatment. *$p < 0.05$, **$p < 0.01$.

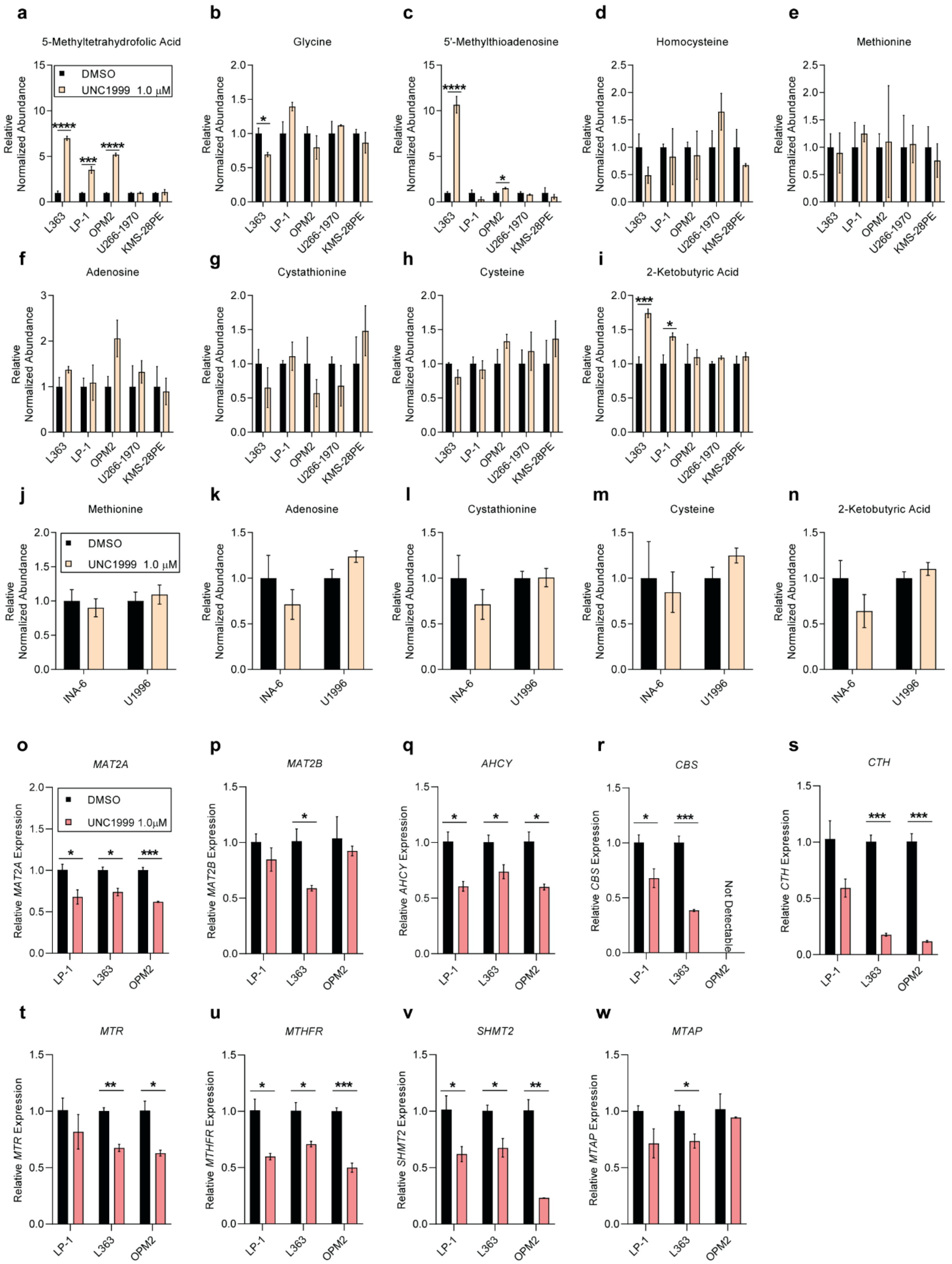

Supplementary Figure 3. UNC1999 treatment has a significant effect on metabolites and genes involved in methionine cycling in UNC1999-responsive MM cell lines. a-i) Relative metabolite abundance as determined by LC-MS for **a)** 5-methyltetrahydrofolic acid, **b)** glycine, **c)** 5'-methylthioadenosine, **d)** homocysteine**, e)** methionine, **f)** adenosine, **g)** cystathionine, **h)** cysteine and **i)** 2-ketobutyric acid. Statistical analysis was performed with multiple t-test. Values: mean with SEM. **o-q)** RT-qPCR analysis of gene expression of (o) *MAT2A*, (p) MAT2B, (q) *AHCY*, (r) *CBS*, (s) *CTH*, (t) *MTR*, (u) *MTHFR*, (v) *SHMT2* and (w) *MTAP*. Statistical analysis was performed with multiple t-test. Values: mean with SEM. $n_{\text{(biological replicates)}}$ = 3 in all figure panels. *p < 0.05, **p < 0.01, ***p < 0.001, ****p < 0.0001

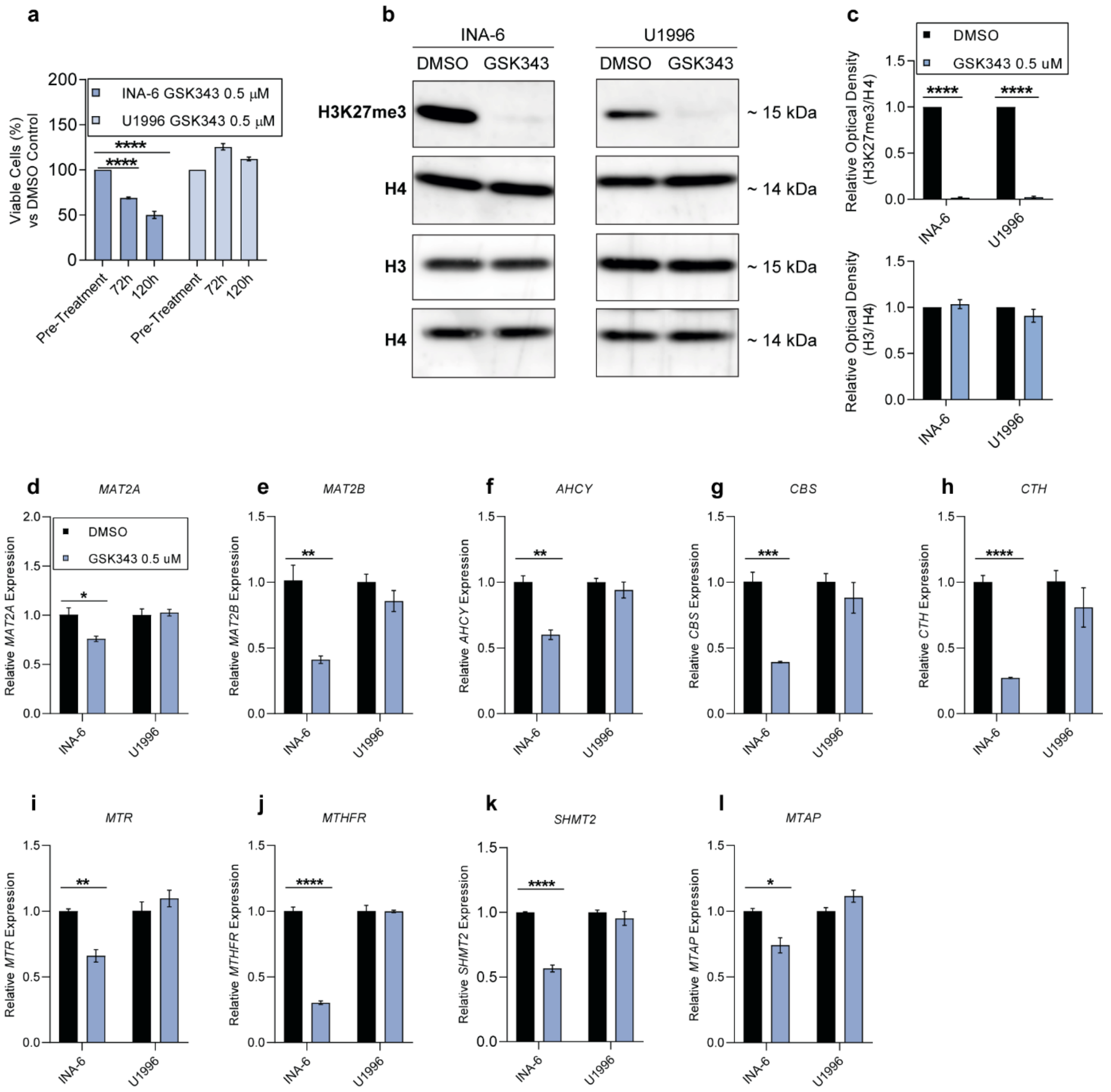

Supplementary Figure 4. Treatment with the EZH2i GSK343 on INA-6 and U1996 cells. a) Viability of INA-6 and U1996 cells treated with 0.5 μM of GSK343. **b)** Western blot against H3K27me3 and H3 in INA-6 and U1996 after UNC1999 treatment. The corresponding uncropped western blots can be found in Supplementary Figure 9f-g. **c)** Signal quantification of the western blots shown in (b). Optical density of H3K27me3 and H3 was normalized against total histone H4. Statistical analysis was performed with two-tailed t-test. Values: mean with SEM. **d-l)** Gene expression analysis via RT-qPCR of all methionine cycling-associated genes in INA-6 and U1996 cells. Statistical analysis was performed with multiple t-test. Values: mean with SEM. $n_{(\text{biological replicates})} = 3$ in all figure panels. *$p < 0.05$, **$p < 0.01$, ***$p < 0.001$, ****$p < 0.0001$.

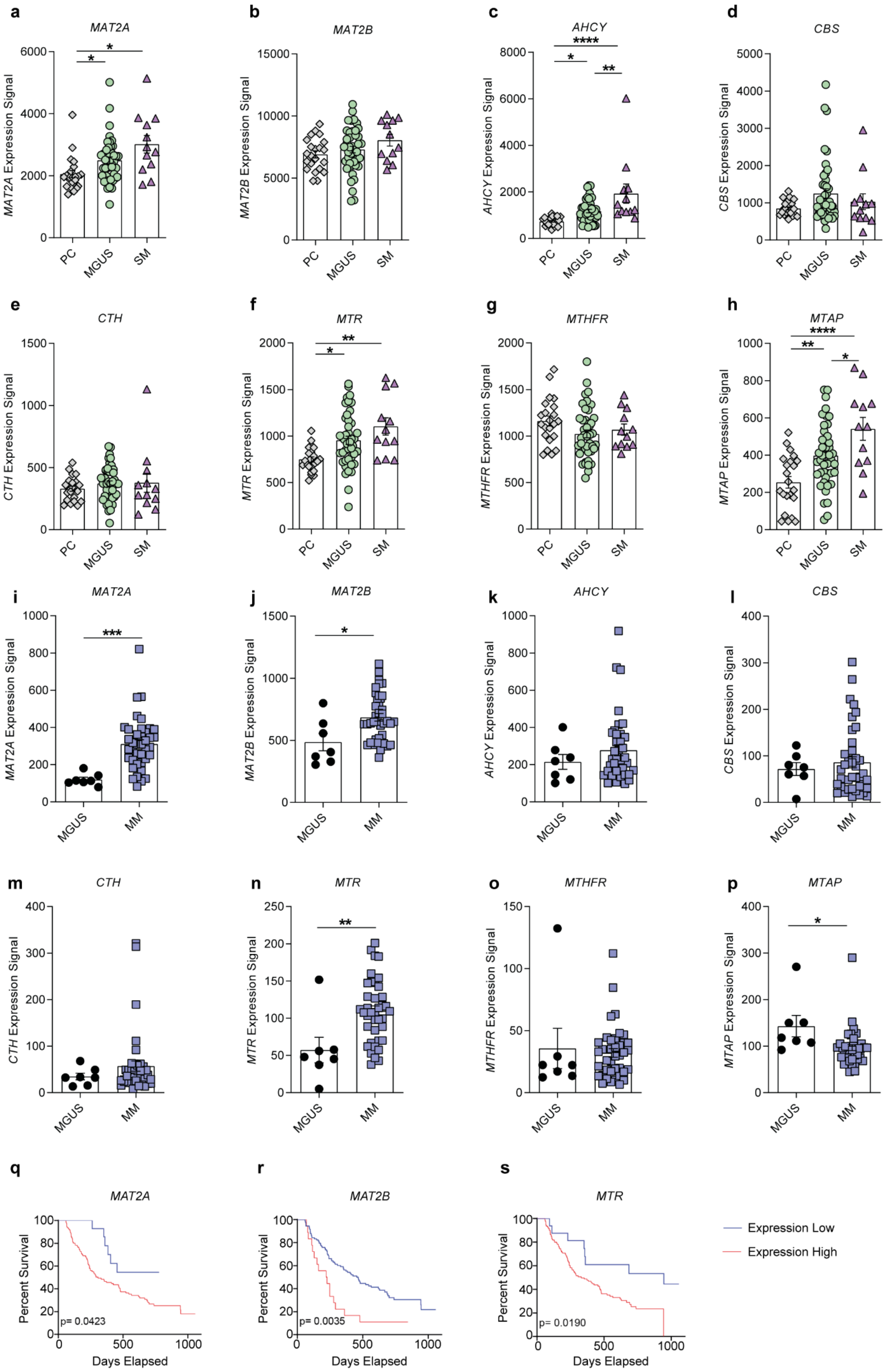

a
MAT2A
MAT2A Expression Signal
PC
MGUS
SM
b
MAT2B
MAT2B Expression Signal
c
AHCY
AHCY Expression Signal
d
CBS
CBS Expression Signal
e
CTH
CTH Expression Signal
f
MTR
MTR Expression Signal
g
MTHFR
MTHFR Expression Signal
h
MTAP
MTAP Expression Signal
i
MAT2A
MAT2A Expression Signal
MGUS
MM
j
MAT2B
MAT2B Expression Signal
k
AHCY
AHCY Expression Signal
l
CBS
CBS Expression Signal
m
CTH
CTH Expression Signal
n
MTR
MTR Expression Signal
o
MTHFR
MTHFR Expression Signal
p
MTAP
MTAP Expression Signal
q
MAT2A
Percent Survival
Days Elapsed
p= 0.0423
r
MAT2B
Percent Survival
Days Elapsed
p= 0.0035
s
MTR
Percent Survival
Days Elapsed
p= 0.0190
Expression Low
Expression High

Supplementary Figure 5. ***In silico*** **analysis of methionine cycling-associated genes in patients with smouldering myeloma (SM), monoclonal gammopathy of undetermined significance (MGUS) and MM. a-h)** Normalized (MAS5) gene expression data of $CD138^+$ cells from SM and MGUS patients for genes associated with methionine cycling, as compared to normal plasma cells (PCs) (U133 Plus 2.0; Affymetrix, GSE5900). Statistics was performed using one-way ANOVA, with Tukey test for multiple comparisons. Values: means with SEM **a-h)** Normalized (MAS5) gene expression data of MM patients for genes associated with methionine cycling, as compared to MGUS (U133 Plus 2.0; Affymetrix, GSE2113). Statistics was performed using two-tailed t-test. Values: means with SEM. **i-p)** Normalized (MAS5) gene expression data of MM patients not responding to bortezomib monotherapy in genes associated with methionine cycling (U133 Plus 2.0; Affymetrix, GSE9782). The analysis was performed on GenomicScape using log-rank test. *p < 0.05, **p < 0.01, ***p < 0.001 ****p < 0.0001.

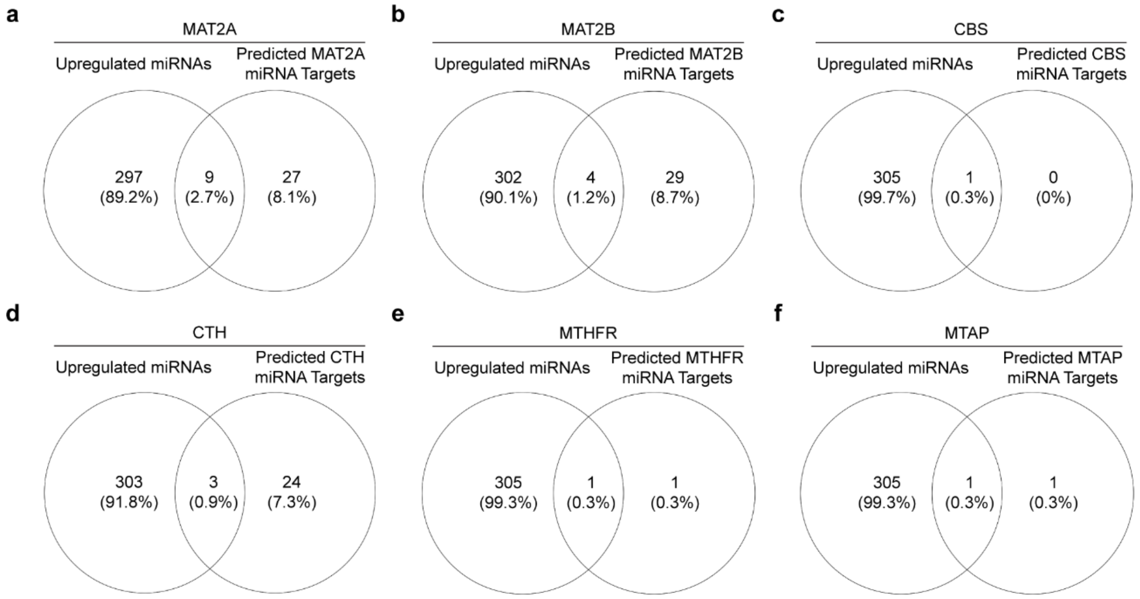

g

**Target Transcript**	**miRNA**
MAT2A	hsa-miR-134-5p
	hsa-miR-23a-3p
	hsa-miR-4306
	hsa-miR-185-5p
	hsa-miR-22-3p
	hsa-miR-4429
	hsa-miR-320c
	hsa-miR-494-3p
	hsa-miR-30d-5p

**Target Transcript**	**miRNA**
MAT2B	hsa-miR-134-5p
	hsa-miR-223-3p
	hsa-miR-454-3p
	hsa-miR-130a-3p

**Target Transcript**	**miRNA**
CTH	hsa-miR-192-5p
	hsa-miR-30d-5p
	hsa-miR-27a-3p

**Target Transcript**	**miRNA**
CBS	hsa-miR-494-3p

**Target Transcript**	**miRNA**
MTHFR	hsa-miR-22-3p

**Target Transcript**	**miRNA**
MTAP	hsa-miR-21-5p

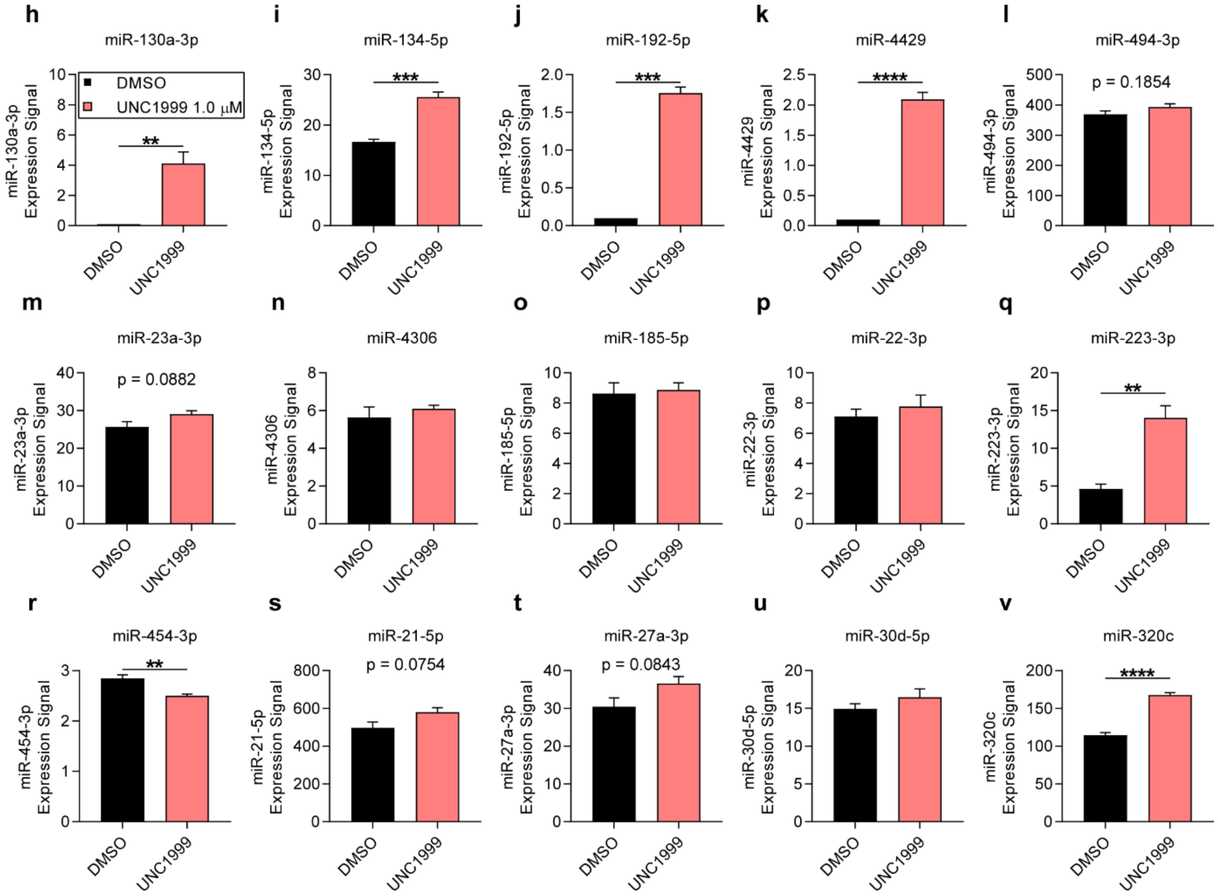

Supplementary Figure 6. 15 miRNAs predicted to target methionine cycling-associated genes were upregulated in INA-6 cells upon UNC1999 treatment. Data on miRNAs upregulated upon UNC1999 treatment were retrieved from the previously published miRNA array GSE:87715[11] and analysed with TargetScanHuman prediction algorithm[28]. **a-f)** Venn diagrams showing the overlap between miRNAs predicted to target methionine cycling-associated genes and all UNC1999-upregulated miRNAs identified in INA-6 cells. The analysis yielded 15 miRNAs that were upregulated in INA-6 cells upon UNC1999 treatment and were predicted to target genes involved in methionine cycling. **g)** Target prediction for each identified miRNA, sorted by target gene**. h-v)** Expression signal of the identified miRNAs (as resulted from the miRNA array). Statistical analysis was performed with two tailed t-test. Values: mean with SEM. $n_{(biological\ replicates)} = 3$ in all figure panels. *$p < 0.05$, **$p < 0.01$, ***$p < 0.001$ ****$p < 0.0001$.

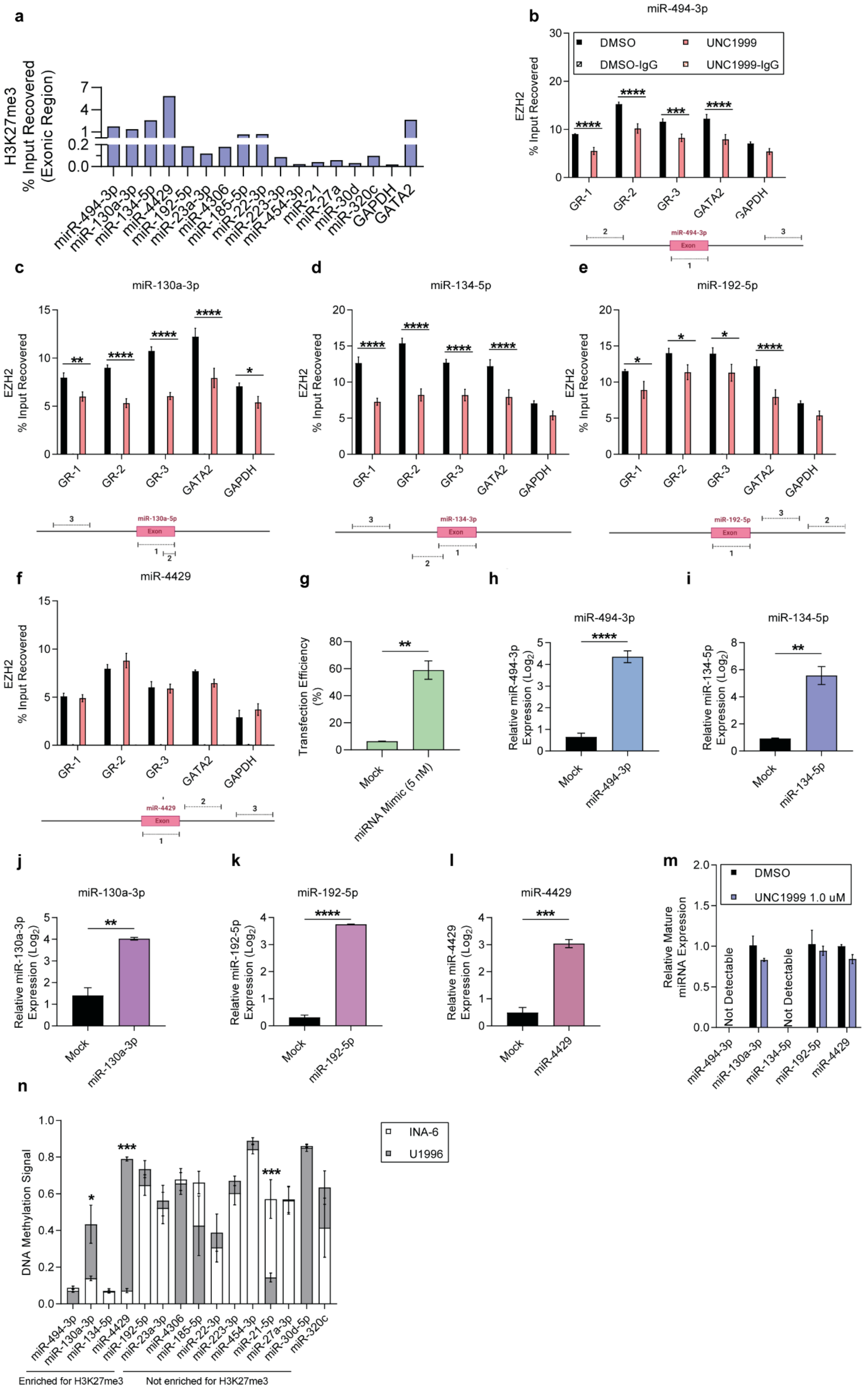

a
H3K27me3 % Input Recovered (Exonic Region)
miR-494-3p
miR-130a-3p
miR-134-5p
miR-4429
miR-192-5p
miR-23a-3p
miR-4306
miR-185-5p
miR-22-3p
miR-223-3p
miR-454-3p
miR-21
miR-27a
miR-30d
miR-320c
GAPDH
GATA2
b
miR-494-3p
DMSO
UNC1999
DMSO-IgG
UNC1999-IgG
EZH2 % Input Recovered
GR-1
GR-2
GR-3
GATA2
GAPDH
Exon
c
miR-130a-3p
d
miR-134-5p
e
miR-192-5p
f
miR-4429
g
Transfection Efficiency (%)
Mock
miRNA Mimic (5 nM)
h
miR-494-3p
Relative miR-494-3p Expression (Log2)
i
miR-134-5p
Relative miR-134-5p Expression (Log2)
j
miR-130a-3p
Relative miR-130a-3p Expression (Log2)
k
miR-192-5p
Relative miR-192-5p Expression (Log2)
l
miR-4429
Relative miR-4429 Expression (Log2)
m
DMSO
UNC1999 1.0 uM
Relative Mature miRNA Expression
Not Detectable
n
DNA Methylation Signal
INA-6
U1996
miR-21-5p
miR-27a-3p
miR-30d-5p
Enriched for H3K27me3
Not enriched for H3K27me3

Supplementary Figure 7. Selection of five UNC1999-upregulated miRNAs that were enriched for H3K27me3. a) ChIP-qPCR of H3K27me3 enrichment in exon regions of fifteen miRNAs predicted to target methionine cycling-associated genes. miR-494-3p, miR-130a-3p, miR-134-5p miR4429, and miR-192-5p were selected as candidates for further analysis, as they were enriched for H3K27me3 **b-f)** EZH2 enrichment in GR-1,2,3 for (b) miRNA-494-3p (c) miR-130a-3p, (d) miR-134-5p and (e) miR-192-5p (f) miR-4429. GR = genomic region. Statistical analysis of all ChIP-qPCR was performed with two-way ANOVA. Values: mean with SEM. **g)** FACS analysis of MCF7 cells transfected with 5 nM of a control miRNA mimic coupled to 5'FAM fluorophore, 48 hours post-transfection. $n_{(biological\ replicates)} = 2$. **h-l)** Taqman RT-qPCR analysis of mimics for (h) miR-494-3p, (i) miR-130a-3p, (j) miR-134-5p, (k) miR-192-5p and (l) miR-4429 expression 48h post-transfection in MCF7 cells. Statistical analysis was performed with two-tailed t-test. Values: mean with SEM. **m)** RT-qPCR analysis of five PRC2-targeted-miRNAs in U1996 cells, post-UNC1999 treatment. Statistical analysis was performed with multiple t-test. Values: mean with SEM. **n)** Enrichment of DNA methylation on CpG sites surrounding the indicated miRNAs in INA-6 and U1996 cells. Statistical analysis was performed with multiple t-test. Values: mean with SEM. $n_{(biological\ replicates)} = 3$ in all figure panels except panel (a) and (g). *$p < 0.05$, **$p < 0.01$, ***$p < 0.001$ ****$p < 0.0001$.

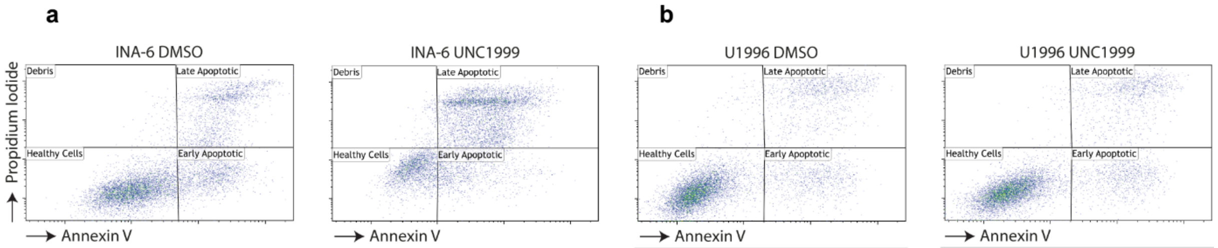

INA-6 DMSO

Gate	Number	%Gated
All	9 003	100,00
Debris	90	1,00
Early Apoptotic	1 654	18,37
Healthy Cells	5 506	61,16
Late Apoptotic	1 753	19,47

INA-6 UNC1999

Gate	Number	%Gated
All	8 573	100,00
Debris	156	1,82
Early Apoptotic	1 396	16,28
Healthy Cells	2 265	26,42
Late Apoptotic	4 756	55,48

U1996 DMSO

Gate	Number	%Gated
All	8 961	100,00
Debris	113	1,26
Early Apoptotic	977	10,90
Healthy Cells	6 643	74,13
Late Apoptotic	1 228	13,70

U1996 UNC1999

Gate	Number	%Gated
All	8 628	100,00
Debris	53	0,61
Early Apoptotic	951	11,02
Healthy Cells	6 526	75,64
Late Apoptotic	1 098	12,73

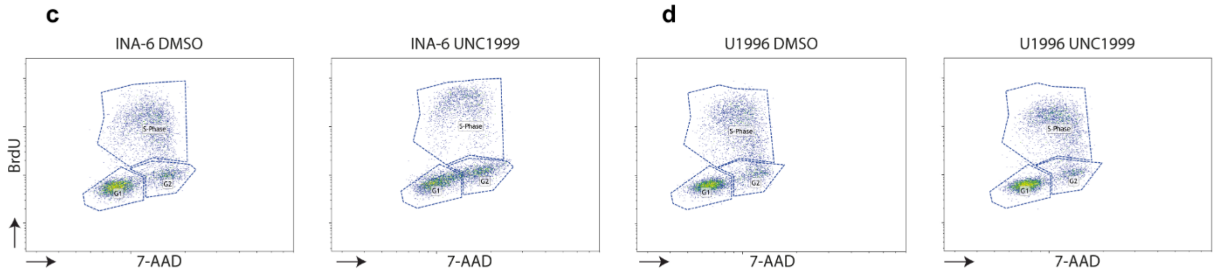

INA-6 DMSO

Gate	Number	%Gated
All	7 700	100,00
G1	3 627	47,10
G2	1 147	14,90
S-Phase	2 780	36,10

INA-6 UNC1999

Gate	Number	%Gated
All	6 571	100,00
G1	2 832	43,10
G2	1 674	25,48
S-Phase	1 870	28,46

U1996 DMSO

Gate	Number	%Gated
All	6 935	100,00
G1	3 196	46,09
G2	972	14,02
S-Phase	2 664	38,41

U1996 UNC1999

Gate	Number	%Gated
All	7 231	100,00
G1	3 581	49,52
G2	815	11,27
S-Phase	2 734	37,81

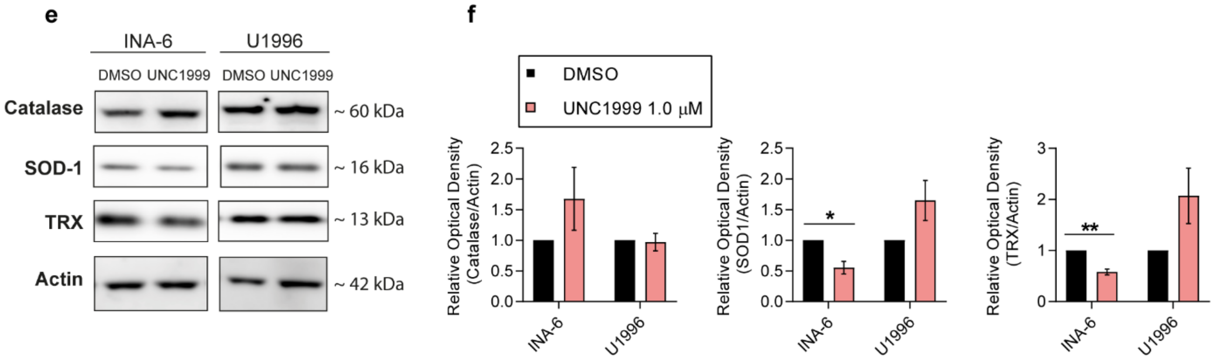

Supplementary Figure 8. UNC1999 treatment triggers G2 arrest, increased apoptotic response and a reduction of ROS defence in INA-6 cells. a-b) Flow cytometry analysis of apoptosis in (a) INA-6 and (b) U1996 cells, stained with Annexin-V-FITC and propidium iodine (PI). **c-d)** Flow cytometry analysis of (c) INA-6 and (d) U1996 cells staining with BrdU and 7-AAD, for evaluation of newly synthesised DNA during cell cycle progression. **e)** Representative western blot against the oxidative defence enzymes Catalase, Superoxide dismutase 1 (SOD1) and Thioredoxin (TRX) in UNC1999-treated INA-6 and U1996 cells. The corresponding uncropped western blot can be found in Supplementary Figure 9e. **f)** Signal quantification of the western blot shown in (e). Optical density of each enzyme was normalized against actin. Statistical analysis was performed with multiple t-test. Values: mean with SEM. $n_{(biological\ replicates)} = 3$ in all figure panels. *$p < 0.05$, **$p < 0.01$, ***$p < 0.001$, ****$p < 0.0001$.

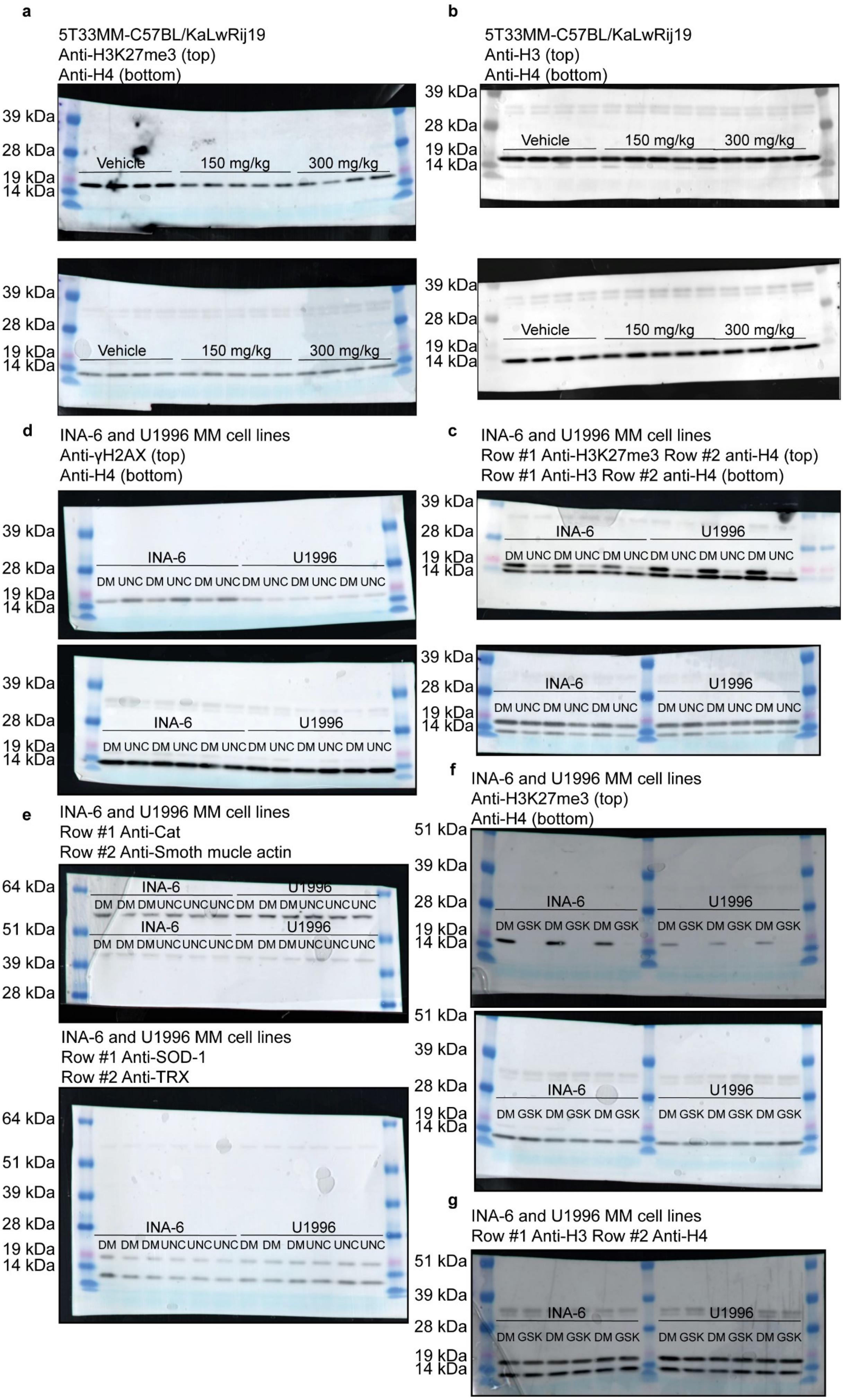

a
5T33MM-C57BL/KaLwRij19
Anti-H3K27me3 (top)
Anti-H4 (bottom)
39 kDa
28 kDa
19 kDa
14 kDa
Vehicle
150 mg/kg
300 mg/kg
39 kDa
28 kDa
19 kDa
14 kDa
Vehicle
150 mg/kg
300 mg/kg
b
5T33MM-C57BL/KaLwRij19
Anti-H3 (top)
Anti-H4 (bottom)
39 kDa
28 kDa
19 kDa
14 kDa
Vehicle
150 mg/kg
300 mg/kg
39 kDa
28 kDa
19 kDa
14 kDa
Vehicle
150 mg/kg
300 mg/kg
d
INA-6 and U1996 MM cell lines
Anti-γH2AX (top)
Anti-H4 (bottom)
39 kDa
28 kDa
19 kDa
14 kDa
INA-6
U1996
DM UNC DM UNC DM UNC DM UNC DM UNC DM UNC
39 kDa
28 kDa
19 kDa
14 kDa
INA-6
U1996
DM UNC DM UNC DM UNC DM UNC DM UNC DM UNC
c
INA-6 and U1996 MM cell lines
Row #1 Anti-H3K27me3 Row #2 anti-H4 (top)
Row #1 Anti-H3 Row #2 anti-H4 (bottom)
39 kDa
28 kDa
19 kDa
14 kDa
INA-6
U1996
DM UNC DM UNC DM UNC DM UNC DM UNC DM UNC
39 kDa
28 kDa
19 kDa
14 kDa
INA-6
U1996
DM UNC DM UNC DM UNC DM UNC DM UNC DM UNC
e
INA-6 and U1996 MM cell lines
Row #1 Anti-Cat
Row #2 Anti-Smoth mucle actin
64 kDa
51 kDa
39 kDa
28 kDa
INA-6
U1996
DM DM DM UNC UNC UNC DM DM DM UNC UNC UNC
INA-6
U1996
DM DM DM UNC UNC UNC DM DM DM UNC UNC UNC
INA-6 and U1996 MM cell lines
Row #1 Anti-SOD-1
Row #2 Anti-TRX
64 kDa
51 kDa
39 kDa
28 kDa
19 kDa
14 kDa
INA-6
U1996
DM DM DM UNC UNC UNC DM DM DM UNC UNC UNC
f
INA-6 and U1996 MM cell lines
Anti-H3K27me3 (top)
Anti-H4 (bottom)
51 kDa
39 kDa
28 kDa
19 kDa
14 kDa
INA-6
U1996
DM GSK DM GSK DM GSK DM GSK DM GSK DM GSK
51 kDa
39 kDa
28 kDa
19 kDa
14 kDa
INA-6
U1996
DM GSK DM GSK DM GSK DM GSK DM GSK DM GSK
g
INA-6 and U1996 MM cell lines
Row #1 Anti-H3 Row #2 Anti-H4
51 kDa
39 kDa
28 kDa
19 kDa
14 kDa
INA-6
U1996
DM GSK DM GSK DM GSK DM GSK DM GSK DM GSK

Supplementary Figure 9. Uncropped and unmodified western blot membranes. a-b) Western blot of (a) H3K27me3 and total (b) histone 3 in UNC1999-treated mice from corresponding Figure 1a. **c-d)** Western blot of (c) H3K27me3 and total histone 3 and (d) γH2AX in INA-6 and U1996 cells, corresponding to images in Figure 2c-d and 5c. **e)** Western blot of the oxidative defence enzymes Catalase, SOD1 and TRX, corresponding to Supplementary Figure 8e. **f-g)** Western blot of (f) H3K27me3 and (g) th3 of GSK343-treated INA-6 and U1996 cells, corresponding to Supplementary Figure 4b. *DM = DMSO, *UNC = UNC1999 and *GSK = GSK343.
